# Identification of Virulence Associated Region during Highly Pathogenic Porcine Reproductive and Respiratory Syndrome Virus during Attenuation In Vitro: Complex Question with Different Strain Backgrounds

**DOI:** 10.3390/v14010040

**Published:** 2021-12-27

**Authors:** Yifeng Jiang, Wu Tong, Lingxue Yu, Liwei Li, Fei Gao, Guoxin Li, Changlong Liu, Pengfei Chen, Qi Shen, Yujiao Zhang, Yanjun Zhou, Guangzhi Tong

**Affiliations:** 1 Shanghai Veterinary Research Institute, Chinese Academy of Agricultural Sciences, Shanghai 200241, China; jiangyifeng@shvri.ac.cn (Y.J.); wutong@shvri.ac.cn (W.T.); yulingxue@shvri.ac.cn (L.Y.); liliwei@shvri.ac.cn (L.L.); feigao@shvri.ac.cn (F.G.); guoxinli@shvri.ac.cn (G.L.); liuchanglong@shvri.ac.cn (C.L.); pfchen@shvri.ac.cn (P.C.); m18019186968@163.com (Q.S.); 18021031162@163.com (Y.Z.); 2 Jiangsu Co-Innovation Center for the Prevention and Control of Important Animal Infectious Disease and Zoonosis, Yangzhou University, Yangzhou 225009, China

**Keywords:** PRRSV, virulence change, reverse genetics system

## Abstract

Highly pathogenic porcine reproductive and respiratory syndrome virus PRRSV (HP-PRRSV) was one of the most devastating diseases of the pig industry, among various strategies, vaccination was one of the most useful tools for PRRS control. Attenuated live vaccine was used worldwide, however, the genetic basis of HP-PRRSV virulence change during attenuation remain to be determined. Here, to identify virulence associated regions of HP-PRRSV during attenuation in vitro, six full-length infectious cDNA clones with interchanges of 5′UTR + ORF1a, ORF1b, and ORF2-7 + 3′UTR regions between HP-PRRSV strain HuN4-F5 and its attenuated vaccine strain HuN4-F112 were generated, and chimeric viruses were rescued. Piglets were inoculated with chimeric viruses and their parental viruses, and rectal temperature were recorded daily, and serum were collected for future experiments. Our results showed that ORF1a played an important role on virus replication, cytokine response and lung damage, the exchange of ORF1b and ORF2-7 in different backbone led to different exhibition on virus replication in vivo/vitro and cytokine response. Among 9 PRRSV attenuated series, consistent amino acid changes during PRRSV attenuation were found in NSP4, NSP9, GP2, E, GP3 and GP4. Our study provides a fundamental data for the investigation of PRRSV attenuation, the different results of the virulence change among different studies indicated that different mechanisms might be used during PRRSV virulence enhancement in vivo and attenuation in vitro.

## 1. Introduction

Porcine reproductive and respiratory syndrome (PRRS) causes significant economic loss to the swine industry worldwide [1]. PRRS is characterized by reproductive failure and stillbirth in sows and respiratory problems in piglets [2,3]. The causative agent, PRRS virus (PRRSV), is an enveloped, single-stranded, positive-sense RNA virus belong to the family Arteriviridae, genus Betaarterivirus [4,5,6,7,8]. The PRRSV genome contains a short 5′ untranslated region (UTR), a 3′ poly-A tail, and at least 10 open reading frames (ORFs). ORF1a and ORF1b encode the nonstructural proteins which processed by self-cleavage into at least 13 nonstructural proteins, involved in viral replication and transcription [9,10,11,12]. ORF2, ORF2a, ORF3, ORF4, ORF5, ORF5a, ORF6, and ORF7 which encode the structural proteins GP2, E, GP3, GP4, GP5, GP5a, M, and N, respectively [10,11,13].

Since PRRS was found in 1990s, vaccines were investigated to control PRRS and only live vaccine could provide fully protection to homologous strain challenge [14,15]. Although many studies revealed partial mechanism of virulence enhancement in natural condition, the mechanism of decrease virulence during vaccine strain attenuation was still unknown [16,17,18,19,20,21]. Considered the difference routes between virulence enhancement and attenuation, the mechanisms used during the two routes might not totally the same, research on the mechanism of attenuation would be useful for vaccine design and improve the understanding of virulence change of PRRSV.

In 2006, a pandemic of highly pathogenic PRRS (HP-PRRS) outbreak in China and resulted significant economic loss to the swine industry [22,23]. Our laboratory isolated and identified a highly pathogenic PRRSV named HuN4 and then an attenuated live vaccine strain was created, named HuN4-F112 [24,25]. In this study, chimeric viruses by interchanging the 5′UTR + ORF1a, ORF1b, and ORF2-7 + 3′UTR regions between HuN4-F5 and HuN4-F112 were generated based on the reverse genetic system of HuN4 and HuN4-F112, and the virulence of each chimeric viruses were tested. 

## 2. Materials and Methods

### 2.1. Cells and Viruses

MARC-145 and BHK-21 cells were cultured in Dulbecco’s Modified Eagle’s medium (DMEM) supplemented with 10% fetal bovine serum (FBS; Invitrogen, Carlsbad, CA, USA) at 37 °C in an atmosphere of 5% CO_2_. Rescued viruses rHuN4-F5 and rHuN4-F112 were developed and maintained by our group [26,27]. 

### 2.2. Construction of Full-Length Chimeric cDNA Clones

**Figure 1:**
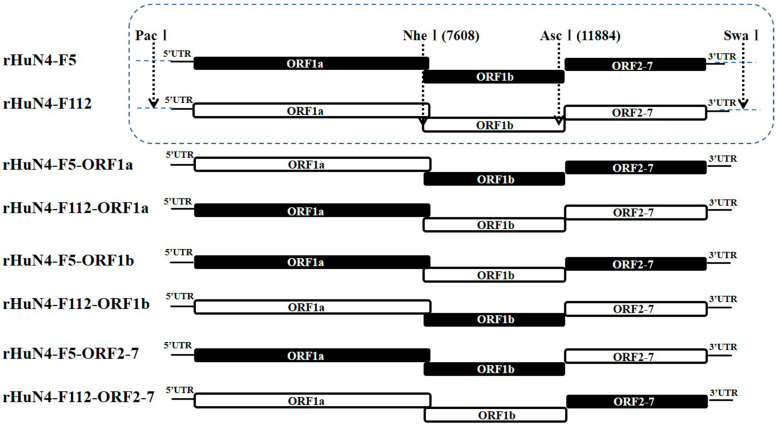
Construction schematic of Chimeric PRRSVs. For the chimeric viruses with backbone of HuN4-F5 or HuN4-F112, *PacI* and *NheI* were used to interchange the 5′UTR + ORF1a region, *NheI* and *AscI* were used to interchange the ORF1b region, *AscI* and *NotI* were used to interchange the ORF2-7 + 3′UTR region. The regions from HuN4-F5 backbone were indicated in black and those from HuN4-F112 backbone were indicated in white.

Full-length HuN4-F5 and HuN4-F112 infectious clones were utilized as backbones to construct chimeric clones. Nucleotide changes incorporated within the genomes of during attenuation of HuN4-F5 and HuN4-F112 were shown in Table S1. *PacI*/*NheI*, *NheI/AscI*, and *AscI/NotI* were used to exchange the 5′UTR + ORF1a, ORF1b, and ORF2-7 + 3′UTR regions between HuN4-F5 and HuN4-F112 (Figure 1). All generated chimeric cDNA clones were confirmed by sequencing.

### 2.3. In Vitro Transcription and Virus Recovery

In vitro transcription and virus recovery were made as described before [26]. Briefly, chimeric full-length cDNA clones were linearized by cleavage with the restriction enzyme *SwaI* downstream from the poly (A) tail, then linearized plasmid DNA were transcribed in vitro according to the procedure of mMessage High Yield Capped RNA Transcription kit (Ambion, Austin, TX, USA). To rescue the viruses, the synthetic RNA was transfected into BHK-21 cells then passaged on MARC-145 cells. The rescued viruses were confirmed using an indirect immunofluorescence assay (IFA) with anti-PRRSV serum and sequencing, and named rHuN4-F5-ORF1a, rHuN4-F5-ORF1b, rHuN4-F5-ORF2-7 (genetic backbone of HuN4-F5 with 5′UTR + ORF1a, ORF1b or ORF2-7 + 3′UTR from HuN4-F112), and rHun4-F112-ORF1a, rHun4-F112-ORF1b, rHun4-F112-ORF2-7 (genetic backbone of HuN4-F112 with 5′UTR + ORF1a, ORF1b, or ORF2-7 + 3′UTR from HuN4-F5), respectively. The 4th passage viruses (P5) on MARC-145 cells were used on the following studies. 

### 2.4. Viral Growth Kinetics

MARC-145 cells were inoculated with 5th passage of the chimeric rescued viruses, rHuN4-F5 and rHuN4-F112 at a MOI of 0.1, respectively. Cell supernatants were collected at 12, 24, 36, 48, 60, 72, 84 and 96 h post infection (hpi). Viral titers were determined on MARC-145 cells and calculated with Reed-Muench method. 

### 2.5. Animal and Experimental Design

Forty-five 40-day-old PRRSV, PCV2, and CSFV antigen free and PRRSV antibody free piglets were selected and randomly divided into nine groups of five piglets each. Piglets in group 1 to 8 were infected with rHuN4-F5-ORF1a, rHuN4-F5-ORF1b, rHuN4-F5-ORF2-7, rHun4-F112-ORF1a, rHun4-F112-ORF1b, rHun4-F112-ORF2-7, rHuN4-F5 and rHuN4-F112 at a dose of 10^5^ TCID_50_ per piglets, intramuscularly. Piglets from group 9 were inoculated with DMEM and served as control. Sera samples were collected at 0, 3, 5, 7, 14, 21, and 28 days post infection (dpi). Rectal temperatures were recorded daily, and clinical feature were observed. Persistent high fever was defined as rectal temperature equal or higher than 40.5 °C and lasting at least 3 days. All animals were euthanized at 28 dpi and necropsied to observe pathological changes of lungs, which were graded using a lung macroscopic score based on the approximate volume that each lung lobe contributed to the entire lung volume [28]. Briefly, the right cranial lobe, right middle lobe, cranial part of the left cranial lobe, and the caudal part of the left cranial lobe each contributed to 10% of the total lung volume, the accessory lobe contributed to 5%, and the right and left caudal lobes each contributed 27.5%. Lung sample which got macroscopic score equal or higher than 50 was defined as severe pathological change, lung sample which got macroscopic score higher than 30 and lower than 50 was defined as medium pathological change, lung sample which got macroscopic score equal or lower than 30 was defined as mild pathological change.

### 2.6. Viral Load Assessment

All sera were submitted to Real-time PCR for viral load detection as described [29]. 

### 2.7. Cytokine Detection

All sera were used to detect IL-1β with the Porcine IL-1 beta Quantikine ELISA Kit (PLB00B; R&D Systems, Inc., Minneapolis, MN, USA); IL-6 with the Porcine IL-6 Quantikine ELISA Kit (P600B; R&D systems, USA); IL-10 with the IL-10 Swine ELISA Kit (KSC0102; Invitrogen, Life Technologies Corporation), and interferon (IFN)-γ with the ProcartaPlex Porcine IFN gamma Simplex Kit (EPX010-660470901; eBioscience Inc., San Diego, CA, USA) according to the manufacturers’ instructions. 

### 2.8. Sequencing Analysis of Nine Series of PRRSV Passage Strains

Nine series of Chinese PRRSV passage strains which could be found in Genebank were selected (Table S1), a single nucleotide (Untranslated region) or amino acid (ORFs) change was recorded, and the mutation ratio were calculate as total changes/length of the region. The nucleotide or amino acid position was based on PRRSV VR2332 strain.

### 2.9. Statistical Analysis

Data are presented as averages ± standard errors (SE). All statistical analyses were performed using SPSS 13.0 statistical software. The results of growth kinetics, virema, cytokine in sera were first analyzed among all the groups, this provided the information about changes of each infectious group compared with control group. Then the results were divided into two combinations, one combination including rHuN4-F5, rHuN4-F5-ORF1a, rHuN4-F5-ORF1b and rHuN4-F5-ORF2-7 groups, the other one including rHuN4-F112, rHuN4-F112-ORF1a, rHuN4-F112-ORF1b and rHuN4-F112-ORF2-7 groups, this provided the detail about influence of each changed region based on the same backbone.

## 3. Results

### 3.1. Construction of Chimeric Clones and Recovery of Viable Chimeric Viruses

Full-length cDNA infectious clones of pHuN4-F5-ORF1a, pHuN4-F5-ORF1b, pHuN4-F5-ORF2-7, pHuN4-F112-ORF1a, pHuN4-F112-ORF1b, and pHuN4-F112-ORF2-7 were constructed. The rescued chimeric viruses (rHuN4-F5-ORF1a, rHuN4-F5-ORF1b, rHuN4-F5-ORF2-7, rHuN4-F112-ORF1a, rHuN4-F112-ORF1b, and rHuN4-F112-ORF2-7) and their parental viruses (rHuN4-F5 and rHuN4-F112) were confirmed by IFA and full-length genome sequencing. 

### 3.2. Viral Growth Kinetics on MARC-145 Cells

**Figure 2:**
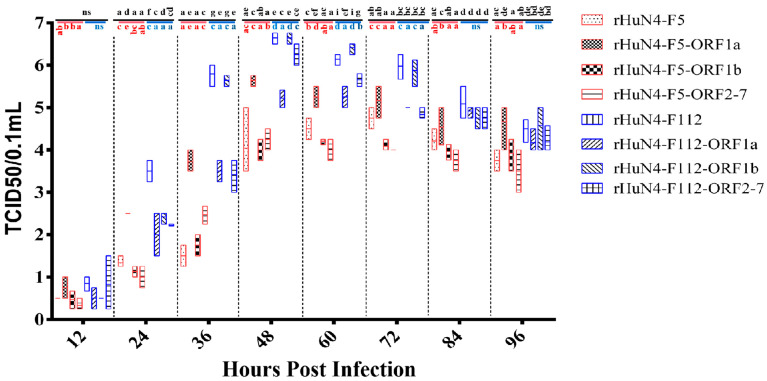
Viral growth kinetics on MARC-145 cells. The growth kinetics of the rescued viruses and their parental virus were determined by assaying the viral titers of the supernatants obtained from 12 to 96 hpi. Chimeric viruses with HuN4 backbone were showed with red border, Chimeric virus with HuN4-F112 backbone were showed with blue border. The results of growth kinetics among all the groups were showed in black alphabets above the black line, the results of rHuN4-F5 and chimeric viruses with rHuN4-F5 backbone were showed in red alphabets below the red line, the results of rHuN4-F5 and chimeric viruses with rHuN4-F5 backbone were showed in blue alphabets below the blue line. Results were showed with single alphabet, or a range of alphabets which were represented by the first and last alphabet. The results which share same alphabet or showed as “ns” means *p* ≥ 0.05; results do not share same alphabet but have adjacent alphabets with each other means *p <* 0.05; results do not share same alphabet or adjacent alphabets with each other means *p <* 0.01. Results among all the viruses were showed with black alphabets above the line, results among HuN4-F5 backbone chimeric viruses were showed with red alphabets below the line, and results among HuN4-F112 backbone chimeric viruses were showed with light blue alphabets below the line.

For the chimeric viruses with HuN4-F5 backbone, compared with rHuN4-F5, significant higher virus titer was found in rHuN4-F5-ORF1a at 24, 36, 60 hpi, and in rHuN4-F5-ORF2-7 at 36 hpi, no significant difference virus titer was found between rHuN4-F5 and rHuN4-ORF1b with the exception of 72 hpi. For the chimeric viruses with HuN4-F112 backbone, significant lower virus titer was found in rHuN4-F112-ORF1a and rHuN4-F112-ORF2-7 at 24, 36, 48, 60, 72 hpi compared with rHuN4-F112, no significant difference virus titer was found between rHuN4-F112 and rHuN4-F112-ORF1b with the exception of 24 hpi. Compared the chimeric viruses which exchange the same region with different backbone, higher virus titer was found in chimeric virus with HuN4-F112 backbone than chimeric virus with HuN4-F5 backbone, with the exception of chimeric viruses which exchange ORF1a. Virus titer of rHuN4-F5-ORF1a and rHuN4-F112-ORF1a had similar virus titer at 36, 48, 60 and 72 hpi (Figure 2).

### 3.3. Rectal Temperature after Infection

**Figure 3:**
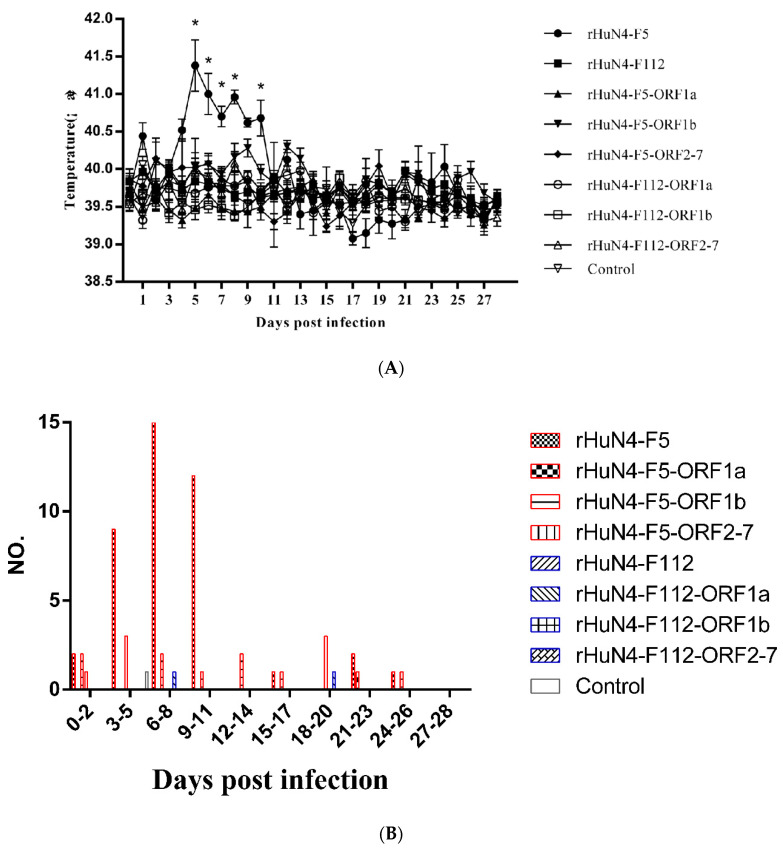

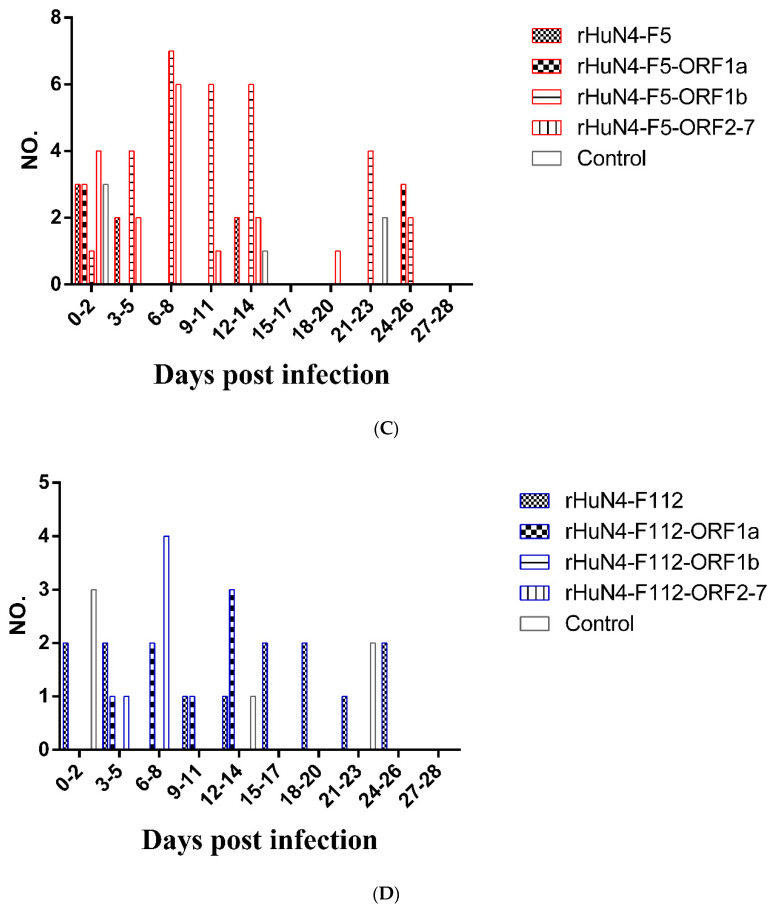
Rectal temperature after infection. (**A**) Mean rectal temperature of each group after infection. (**B**) Amount case of rectal temperature equal or higher than 40.5 °C of each group. (**C**) Amount case of rectal temperature higher than 40 °C and lower than 40.5 °C of groups which infected with HuN4-F5 backbone chimeric viruses. (**D**) Amount case of rectal temperature higher than 40 °C and lower than 40.5 °C of groups which infected with HuN4-F112 backbone chimeric viruses. Mean rectal temperature were presented as means ± standard deviations (error bars), “*” means *p* ≤ 0.5. Cases were counted every 3 days.

After infection, piglets infected with rHuN4-F5 manifested various diseases, including persistent high fever, anorexia, and two piglets died, persistent high fever was found in piglets belonged to rHuN4-F5 group from 4 to 10 dpi, temporary high fever (higher than 40.5 °C) was found in other groups (Figure 3A,B). Then numbers of rectal temperature which higher than 40 °C but lower than 40.5 °C (defined as fever) of each group were counted, more piglets showed fever in rHuN4-F5-ORF1b group than piglets in the other HuN4-F5 backbone chimeric virus groups (Figure 3C); for HuN4-F112 backbone chimeric virus groups, several piglets in rHuN4-F112, rHuN4-ORF1a and rHuN4-ORF2-7 groups showed fever during the test (Figure 3D).

### 3.4. Lung Macroscopic Scores after Infection

Although no significant difference was found among the groups, lungs infected with rHuN4-F5 exhibited serious pathological changes, 40% lung samples got severe pathological change and 60% lung samples got medium pathological change, all the piglets infected with rHuN4-F112 exhibited mild pathological change in lungs (Table 1). For the chimeric viruses which with backbone of HuN4-F5, all the lung samples in rHuN4-F5-ORF2-7 group showed mild pathological change, group rHuN4-F5-ORF1a and rHuN4-F5-ORF1b each showed three samples with medium pathological change (Table 1). For the chimeric viruses which with backbone of HuN4-F112, 4 lungs showed mild pathological change and 1 lung showed severe pathological change in rHuN4-F112-ORF2-7 group, there was 1 lung showed severe and 1 lung showed medium pathological change in both rHuN4-F112-ORF1a and rHuN4-F112-ORF1b groups (Table 1).

### 3.5. Virus Copies in Sera

**Figure 4:**
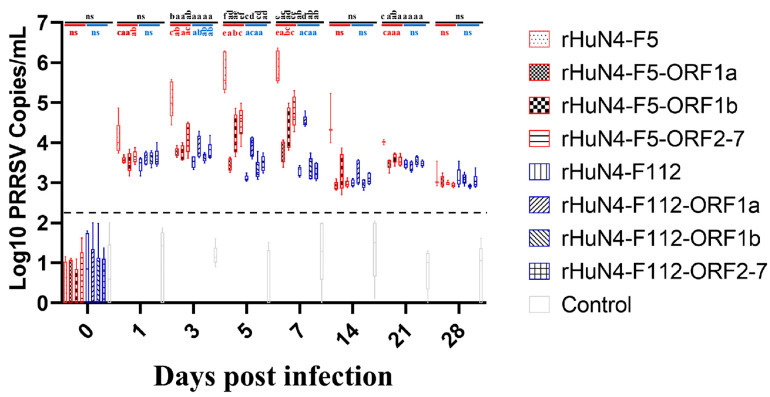
Serum viral copies of each group. The serum viral copies of the rescued viruses and their parental virus were showed with means ± standard deviations. Chimeric viruses with HuN4 backbone were showed with red border, chimeric virus with HuN4-F112 backbone were showed with blue border. The results of serum viral copies among all the groups were showed in black alphabets above the black line, the results of rHuN4-F5 and chimeric viruses with rHuN4-F5 backbone were showed in red alphabets below the red line, the results of rHuN4-F5 and chimeric viruses with rHuN4-F5 backbone were showed in blue alphabets below the blue line. Results were showed with single alphabet, or a range of alphabets which were represented by the first and last alphabet.The results which share same alphabet or showed as “ns” means *p* ≥ 0.05; results do not share same alphabet but have adjacent alphabets with each other means *p* < 0.05; results do not share same alphabet or adjacent alphabets with each other means *p* < 0.01. Results among all the viruses were showed with black alphabets above the line, results among HuN4-F5 backbone chimeric viruses were showed with red alphabets below the line, and results among HuN4-F112 backbone chimeric viruses were showed with light blue alphabets below the line.

Significant higher virus copies were found in group rHuN4-F5 compared with other groups at 3 (with the exception of rHuN4-F5-ORF2-7 group), 5 and 7 dpi (Figure 4). For the chimeric viruses with the same exchange region, significant difference of virus copies was found between rHuN4-F5-ORF2-7 and rHuN4-F112-ORF2-7 at 5 and 7 dpi, rHuN4-F5-ORF1a and rHuN4-F112-ORF1a at 7 dpi (Figure 4). For the chimeric viruses with HuN4-F5 backbone, compared with rHuN4-F5, the change of each region decreased the virus copies dramatically at 1, 3, 5, 7 and 21 dpi (Figure 4). Among the chimeric viruses with HuN4-F5 backbone, rHuN4-F5-ORF2-7 got the higher viral copies at 3, 5 and 7 dpi, rHuN4-F5-ORF1a got the lowest virus copies at 5 and 7 dpi (Figure 4). For the virus with HuN4-F112 backbone, rHuN4-F112-ORF1a got significant higher virus copies at 5 and 7 dpi, no significant difference was found among the other viruses (Figure 4).

### 3.6. Results of Cytokine Detection

**Figure 5:**
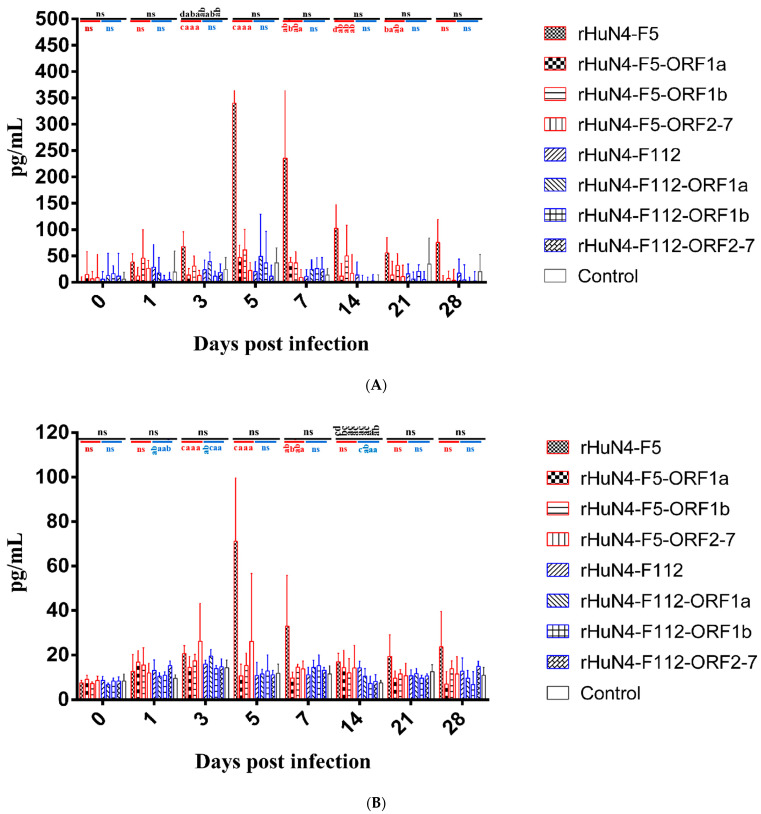

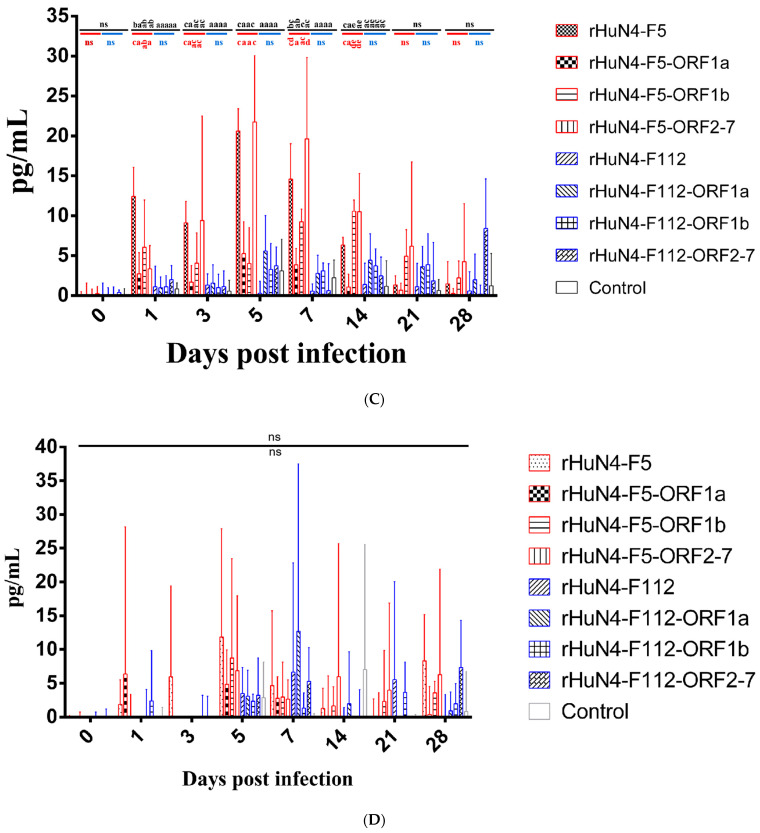
Cytokine response of each group. (**A**) IL-1β response in peripheral blood after infection. (**B**) IL-6 response in peripheral blood after infection. (**C**) IL-10 response in peripheral blood after infection. (**D**) IFN-γ response in peripheral blood after infection. Chimeric viruses with HuN4 backbone were showed with red border, Chimeric virus with HuN4-F112 backbone were showed with blue border. The results of cytokines among all the groups were showed in black alphabets above the black line, the results of rHuN4-F5 and chimeric viruses with rHuN4-F5 backbone were showed in red alphabets below the red line, the results of rHuN4-F5 and chimeric viruses with rHuN4-F5 backbone were showed in blue alphabets below the blue line. Results were showed with single alphabet, or a range of alphabets which were represented by the first and last alphabet. The results which share same alphabet or showed as “ns” means *p* ≥ 0.05; results do not share same alphabet but have adjacent alphabets with each other means *p* < 0.05; results do not share same alphabet or adjacent alphabets with each other means *p* < 0.01. Results among all the viruses were showed with black alphabets above the line, results among HuN4-F5 backbone chimeric viruses were showed with red alphabets below the line, and results among HuN4-F112 backbone chimeric viruses were showed with light blue alphabets below the line.

Among all the groups, the level of IL-1β in response to rHuN4-F5 was significantly higher than the other viruses at 3 dpi, and among the chimeric viruses with backbone HuN4-F5, higher IL-1βwas found in in rHuN4-F5 group at 3, 5, 14, 21 and 28 dpi, there were no significant differences among the chimeric viruses with backbone HuN4-F112 (Figure 5A). 

Higher IL-6 expression was found in rHuN4-F5 group than the other groups at 5, 7, 21 and 28 dpi, but with no statistical difference (Figure 5B). For the chimeric viruses with HuN4-F112 backbone, significant higher IL-6 was found in rHuN4-F112-ORF1a and rHuN4-F112 at 3 and 14 dpi, respectively (Figure 5B). 

Among all the groups, higher IL-10 response to rHuN4-F5 and rHuN4-F5-ORF2-7 were found at 1, 3, 5, 7 and 14 dpi than the other groups (Figure 5C). For the chimeric viruses with HuN4-F5 backbone, significant lower IL-10 response was found in rHuN4-F5-ORF1a group at 1, 3, 5, 7 and 14 dpi compared with rHuN4-F5 group, at 5, 7 and 14 dpi compared with rHuN4-F5-ORF2-7 group (Figure 5C). Compared with other HuN4-F112 backbone chimeric viruses, rHuN4-F112 showed lower IL-10 level in sera, but with no statistical difference (Figure 5C). 

Piglets infected with different rescued viruses exhibit different IFN-γ response and no significant difference was found among the groups (Figure 5D).

### 3.7. Analysis of Genome Change among Nine Series of PRRSV Strain

**Figure 6:**
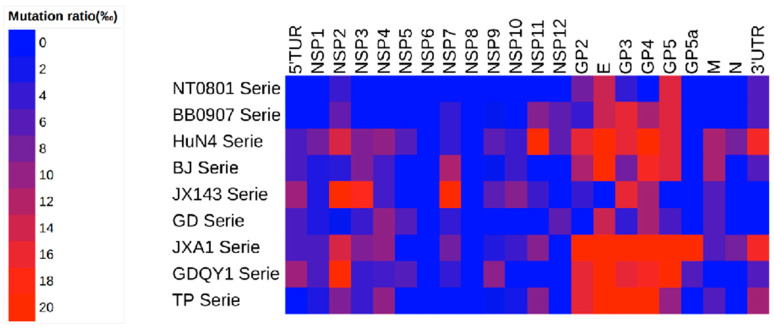
Heat map of mutation during PRRSV attenuation in vitro of each series.

As showed in Figure 6 the most hypervariable region of PRRSV genome during attenuation were NSP2, ORF2, ORF2a, ORF3, ORF4 and ORF5. Compared the whole genome, several consistent change were found, Y92H in NSP4 was found in GD, TP and BB0907 series; P274S in NSP9 was found in GDQY1, TP and BB0907 series; Y50S or Y50F in GP2 was found in HuN4, JXA1, GDQY1 and TP series; I118V or I118T in GP2 was found in HuN4, NT0801, JXA1, GDQY1, BJ and TP series; D9N, D9H or D9Y in E was found in HuN4, NT0801, JXA1,GD, GDQY1, BJ, TP and BB0907 series; L48F in E was found in HuN4, JXA1, GDQY1, BJ and TP series; H79Y in GP3 was found in JXA1, JX143, GDQY1 and BB0907 series; I124V or I124T in GP4 was found in HuN4, JXA1, GD and BJ series (Table S1).

## 4. Discussion

As one of the most important swine diseases, PRRS cause huge economic lose to swine industry worldwide. Immunization should be the most effective and economical tool to control PRRS, but the disadvantage of PRRS vaccine especially virulence revertion not only cause PRRS outbreak but also restricting the use of vaccine, however the mechanism of virulence revertion still unknown [30]. Since it was first isolated in 1990s, the virulence of PRRSV changed continuously [22,23,31,32]. Although many studies focus on the virulence change of PRRSV, but the model of these studies were based on the natural low virulent strain and virulent strain [19,20,21,33,34]. The results of these studies exhibited complex results, NSP1β, NSP2, NSP10, ORF2, ORF3, ORF5 and ORF6 were considered for attenuation, however, another study believe NSP2 may influence the growth attenuated in swine [19,33]. Use FL12 infectious clone, the virulence determinants were NSP1-3, NSP10-12 and OFR2, but use the Chinese HP-PRRSV strain this was NSP9 and NSP10 [20,35]. Our results show all the three regions were contribute to the attenuation which consisted with provide study that 5′UTR/ORF1 and/or structure proteins/3′UTR might be virulence determinants [21]. Considered the difference routes of virulence enhancement in vivo and vaccine attenuation in vitro, study based on the vaccine strain and its parent strain might provide more precise data which could explain the mechanism of vaccine attenuation, and more over provide some hints to the investigation of virulence revertion.

Two HP-PRRSV infectious clones rHuN4-F5 and rHuN4-F112 were used in this study to identify the molecular mechanisms responsible for the virulence change between HuN4-F5 and its attenuated vaccine strain HuN4-F112, this provides an ideal model which could avoid the influence by the difference of virus backbone. 

First, we investigate the influence of in vitro growth kinetics by the exchange of different regions, this might reflect the adaptation and/or replication change of the chimeric viruses on MARC-145 cells, because increasing adaptation on MARC-145 cells was a characteristic during PRRSV attenuation. As shown in Figure 2 the exchange of 5′UTR + ORF1a decrease the growth kinetics of rHuN4-F112 dramatically, and in contrast higher growth kinetics were found in rHuN4-F5-ORF1a compared with rHuN4-F5, considered the function of un-translate region, these results indicated that ORF1a may contribute to the adaptation or replication of PRRSV on MARC-145 cells. Moreover, the exchange of ORF2-7 + 3′ UTR also influence the growth kinetics of PRRSV, although the influence was weaker than ORF1a + 5′ UTR. Between the chimeric viruses with the same exchange regions, virus with backbone of HuN4-F112 got higher growth kinetics than HuN4 backbone viruses with the exception of ORF1a, rHuN4-F5-ORF1a and rHuN4-F112-ORF1a had similar replication level on MARC-145 cells, this indicated ORF1a may played a major role during PRRSV replicate on MARC-145 cells, and this was consisted with provide studies that Nsp2, Nsp3-8 and other parts of the non-structure proteins influence the replication of PRRSV [16,19,20,33,34].

Detection of viremia showed an inverted result compared with growth kinetics which HuN4-F5 backbone viruses got higher viremia than HuN4-F112 backbone viruses with the exception of rHuN4-F5-ORF1a and rHuN4-F112-ORF1a. Significant lower viremia were found in group rHuN4-F5-ORF1a, rHuN4-F5-ORF1b and rHuN4-F5-ORF2-7 compared with rHuN4-F5, the lowest viremia were found in group rHuN4-F5-ORF1a at 5 and 7 dpi. In contrast, among the chimeric viruses with HuN4-F112 backbone, highest viremia were found in group rHuN4-F112-ORF1a at 5 and 7 dpi, but the change of ORF1b and ORF2-7 did not influence the viremia dramatically. The results of viremia and growth kinetics all showed that 5′UTR + ORF1a may contribute to the adaption or replication of PRRSV, and considered the function of un-transcribed region and non-structural protein, the influence may be due to the replication ability change of PRRSV and mainly contribute by ORF1a. These results were consisted with previous study which several Non-Structure Protein coding region may influence the replication of PRRSV both in vivo and vitro [16,20,21,33]. 

Persistent high fever was one of the most important symbols of HP-PRRSV infection, after infection, persistent high fever was only found in group rHuN4-F5, this result indicate that all the three parts of PRRSV genome may influence the virulence of PRRSV in vivo. The results of the present study were consistent with those of other studies reporting that 3′UTR, Nsp1-2, Nsp3-8, Nsp10-12, ORF2, ORF5, ORF6 and 3′UTR were virulence determinants [19,20,21,33,36]. Analysis of the dissected lung tissues also showed that ORF1a, ORF1b, and ORF2-7 may influence the extent of pathological changes, as determined by the macroscopic scores. For the chimeric viruses with HuN4 backbone, more cases of high fever (above 40 °C and below 40.5 °C) were found in group rHuN4-F5-ORF1b which indicate the less influence of ORF1b during virulence change, this was consisted with HuN4-F112 backbone, which rHuN4-F112-ORF1b got less cases of high fever than the other chimeric viruses. Our results were not consisted with provide study which Nsp9 and Nsp10 may contributed to virulence between Classical PRRSV and HP-PRRSV, however this difference may be due to the different virus model of the studies, and indicated that the mechanism of virulence change may different between virulence enhancement in vivo and attenuation in vitro [17,18,35].

PRRSV virulence is determined by many factors, especially the interaction between the virus and host immune system. We found that the cytokines associated with immune response including IL-1β, IL-6, IL-10, and IFN-γ, where IL-1β and IL-6 indicated an acute inflammatory reaction, IL-10 was associated with immune suppression, and IFN-γ was a major antiviral cytokine [37,38,39,40]. 

In group rHuN4-F5, IL-1β and IL-6 levels were significantly higher than the other groups, no significant difference was found among the chimeric viruses with the exception of rHuN4-F5-ORF1b at 3 dpi, indicate that the three regions may all contributed to virulence change. Many groups reported that PRRSV may upregulate IL-10 expression. However, in our study, IL-10 levels varied, the exchange of ORF1a and ORF1b of HuN4 dramatically decrease the IL-10 expression, reverse results were found in HuN4-F112 backbone chimeric viruses which rHuN4-F112-ORF1a and rHuN4-F112-ORF1b induce higher IL-10 expression compared with rHuN4-F112 although no statistic difference was found. These results of ORF1a influence the IL-10 expression consisted with provide studies which Nsp1–2 and Nsp3–8 regions to induce IL-10 production [41,42]. The decrease expression of IL-10 of rHuN4-F5-ORF1b infection may be due to the role of Nsp9 and Nsp10 which were the core components of the membrane-associated viral replication and transcription complex and influence virus replication [43,44]. Although some groups have reported that the GP5 and N proteins may induce IL-10 production during PRRSV infection, our results indicated that ORF2-7 plays a minor role in IL-10 production [45,46]. Low levels of IFN-γ during PRRSV infection has been reported by many groups, in our study, IFN-γ expression varied, this mainly due to the individual difference of piglets, however low level of IFN-γ may reflect the inefficient host immune response [47,48,49].

The reverse results of cytokine response during rHuN4-F5-ORF1a and rHuN4-F112-ORF1a were consisted with in vivo and in vitro virus replication of the two viruses, this indicated that the influence of the exchange of 5′UTR + ORF1a may be mainly due to virus replication which consisted with provide studies that many regions including NSP2 influence the replication of chimeric virus [16,20,36]. However, in this study, the exchange of ORF1b and ORF2-7 + 3′UTR did not got the complete opposite results between rHuN4-F5 and rHuN4-F112, showed that the attenuation of PRRSV in vitro may not consist with virulence increase in vivo. Then we compared the difference amino acid between HuN4-F5 and HuN4-F112, to fully understand the mechanism of attenuation, the other 8 series of viruses were chosen. For all the serious, mutations or deletions were found in the whole genome with the exception of NSP6 and NSP8 (Table S1). Although high quantity amino acid change was found in non-structure protein coding region especially NSP2, high frequency of mutation ratio was found in miner structure protein region. Within the mutations, consistent change was found in NSP4, NSP9, GP2, E, GP3 and GP4. Lots of genome comparison studies use different PRRSV strains to find amino acid which might contribute to virulence change, but results were different with each other, a recent study showed that 519 and 544 amino acid in NSP9 may contribute to virulence change between classical PRRSV and HP-PRRSV, in our study the consistent change in NSP9 was position 274 this also demonstrated the difference during virulence increase in vivo and attenuation in vitro, this may because the change of amino acid during attenuation did not influence the function of NSP9-NSP12, the attenuation may mainly due to the change of cell adaption, however the precise mechanism of PRRSV attenuation need further investigation [17,18,20,21]. Most consistent changes were found in GP2-GP4, which reported as the key proteins interact with CD163 during PRRSV entrance, indicated that the attenuation of PRRSV may at least partly due to the change of cell adaption on MARC-145 cells [50,51]. To conclusion, all the three parts of PRRSV genome were contribute to PRRSV virulence during PRRSV attenuation and ORF1b got less infection on PRRSV virulence change compared with other two regions. The influence of ORF1a on virulence change may due to its influence on PRRSV replication, ORF1b and ORF2-7 may play different roles during PRRSV virulence enhancement and attenuation, and the consist amino acid change among the PRRSV viruses showed that minor protein might contribute to PRRSV attenuation, and indicated that different mechanisms might be used during PRRSV virulence enhancement in vivo and attenuation in vitro.

## Figures and Tables

**Table 1:** Mean values (±SD) of macroscopic scores of lung tissues after infection.

Designation	Number	Macroscopic (Lung)			
		Mean ± SD	Pathological Changes (Score)		
			≤30	30 to 50	≥50
rHuN4-F5	5	65 ± 27.36	0	3	2
rHuN4-F5-ORF1a	5	30.8 ± 14.72	2	3	0
rHuN4-F5-ORF1b	5	33.4 ± 17.08	2	3	0
rHuN4-F5-ORF2-7	5	20 ± 15.03	5	0	0
rHuN4-F112	5	17.6 ± 11.15	5	0	0
rHuN4-F112-ORF1a	5	36.8 ± 21.11	3	1	1
rHuN4-F112-ORF1b	5	41.6 ± 19.76	3	1	1
rHuN4-F112-ORF2-7	5	27.4 ± 14.19	4	0	1
Control	5	15.2 ± 13.92	5	0	0

## Data Availability

Not applicable.

## Supplementary Materials

The following supporting information can be downloaded at: https://www.mdpi.com/article/10.3390/v14010040/s1, Table S1: Information of PRRSV strains and genome change during in vitro passage.
