# Supplementary material for: Identification of Virulence Associated Region during Highly Pathogenic Porcine Reproductive and Respiratory Syndrome Virus during Attenuation In Vitro: Complex Question with Different Strain Backgrounds
Source: Viruses. 2021 Dec 27;14(1):40. doi: 10.3390/v14010040 (PMC8780124; doi:10.3390/v14010040)
Supplement: Supplementary file 1 [file viruses-14-00040-s001.zip › viruses-1531607-supplementary.pdf]

Table S1. Information of PRRSV strains and genome change during *in vitro* passage.

Series	Name	NO.	5′UTR						NSP1										
			59	118	119	137	163	166	108	163	187	200	204	240	257	266	281	287	329
	VR2332	EF536003	T	G	A	A	C	T	A	P	G	S	R	F	H	P	E	I	Y
HuN4	HuN4	EF635006	C	G	G	A	C	T	A	P	G	S	R	F	C	P	K	I	Y
	HuN4-F40	-	T	-	-	-	-	-	-	-	-	S	-	-	H	-	E	-	-
	HuN4-F60	-	T	-	-	-	-	-	-	-	-	F	-	-	H	-	E	-	-
	HuN4-F112	-	T	-	-	-	-	-	-	-	-	F	-	-	H	-	E	-	-
NT0801	NT0801	HQ315836	T	-	-	-	-	-	-	-	-	-	-	-	H	-	E	-	-
	NT0801-P10	KJ523894	T	-	-	-	-	-	-	-	-	-	-	-	H	-	E	-	-
	NT0801-P30	KJ523895	T	-	-	-	-	-	-	-	-	-	-	-	H	-	E	-	-
	NT0801-P50	KJ523896	T	-	-	-	-	-	-	-	-	-	-	-	H	-	E	-	-
	NT0801-P80	KJ523897	T	-	-	-	-	-	-	-	-	-	-	-	H	-	E	-	-
JXA1	JXA1	EF112445	T	-	G	-	-	-	-	-	D	-	-	F	H	-	E	-	-
	JXA1 P10	FJ548854	T	-	G	-	-	-	-	-	G	-	-	S	H	-	E	-	-
	JXA1 P15	FJ548855	T	-	G	-	-	-	-	-	G	-	-	S	H	-	E	-	-
	JXA1 P45	FJ548851	T	-	G	-	-	-	-	-	G	-	-	F	H	-	E	-	-
	JXA1 P70	FJ548852	T	-	G	-	-	-	-	-	G	-	-	F	H	-	E	-	-
	JXA1 P80	FJ548853	T	-	G	-	-	-	-	-	G	-	-	F	H	-	E	-	-
	JXA1 P100	KC422725	T	-	G	-	-	-	-	-	G	-	-	F	H	-	E	-	-
	JXA1 P110	KC422726	T	-	G	-	-	-	-	-	G	-	-	F	H	-	E	-	-
	JXA1 P120	KC422727	T	-	G	-	-	-	-	-	G	-	-	F	H	-	E	-	-
	JXA1-P130	KC422728	T	-	G	-	-	-	-	-	G	-	-	F	H	-	E	-	-
	JXA1-P140	KC422729	T	-	Del	-	-	-	-	-	G	-	-	F	H	-	E	-	-
	JXA1-P150	KC422730	T	-	Del	-	-	-	-	-	G	-	-	F	H	-	E	-	-
	JXA1-P160	KC422731	T	-	Del	-	-	-	-	-	G	-	-	S	H	-	E	-	-
	JXA1-P170	JQ804986	T	-	Del	-	-	-	-	-	G	-	-	F	H	-	E	-	-
JX143	JX143	EU708726	T	-	-	-	T	T	P	-	-	-	-	-	Y	-	E	-	-
	JXM20	GQ499193	T	-	-	-	C	C	A	-	-	-	-	-	Y	-	E	-	-
	JXM40	GQ499194	T	-	-	-	C	C	A	-	-	-	-	-	Y	-	E	-	-

	JXM60	GQ499195	T	-	-	-	C	C	A	-	-	-	-	-	Y	-	E	-	-
	JXM80	GQ499196	T	-	-	-	C	C	A	-	-	-	-	-	Y	-	E	-	-
	JXM100	GQ475526	T	-	-	-	C	C	A	-	-	-	-	-	Y	-	E	-	-
GD	GD	EU825724	T	-	-	A	-	-	-	-	-	-	-	-	Y	-	E	-	Y
	GD-P100	GU143913	T	-	-	G	-	-	-	-	-	-	-	-	Y	-	E	-	H
GDQY1	GDQY1	JN387271	T	G	G	-	-	-	-	H	-	-	-	-	H	P	E	-	-
	GDQY1VP65	JN387272	T	T	Del	-	-	-	-	P	-	-	-	-	H	P	E	-	-
	GDQY1VP80	JN387273	T	T	Del	-	-	-	-	P	-	-	-	-	H	L	E	-	-
	GDQY1VP100	JN387274	T	T	Del	-	-	-	-	P	-	-	-	-	H	L	E	-	-
BJ	BJ	EU825723	T	-	-	-	-	T	-	-	-	-	R	-	Y	-	E	-	-
	BJ-F20	KP890337	T	-	-	-	-	T	-	-	-	-	R	-	Y	-	E	-	-
	BJ-F40	KP890338	T	-	-	-	-	T	-	-	-	-	R	-	Y	-	E	-	-
	BJ-F80	KP890340	T	-	-	-	-	C	-	-	-	-	R	-	Y	-	E	-	-
	BJ-F100	KP890341	T	-	-	-	-	T	-	-	-	-	C	-	Y	-	E	-	-
	BJ-F150	KP890342	T	-	-	-	-	C		-	-	-	C	-	Y	-	E	-	-
TP	TP	EU864233	T	-	-	-	-	-	-	-	-	-	-	-	H	-	E	I	-
	TP_P60	GU232736	T	-	-	-	-	-	-	-	-	-	-	-	H	-	E	T	-
	TP_P90	GU232737	T	-	-	-	-	-	-	-	-	-	-	-	H	-	E	I	-
BB0907	BB0907	HQ315835	T	-	-	-	-	-	-	-	-	-	-	-	-	-	E	-	-
	BB0907-s34	KM453698	T	-	-	-	-	-	-	-	-	-	-	-	-	-	E	-	-
	BB0907-F44	KM453699	T	-	-	-	-	-	-	-	-	-	-	-	-	-	E	-	-
Series	Name	NO.	NSP2																
			3	19	23	24	30	45	63	81	84	95	100	131	160	189	190	195	214
	VR2332	EF536003	G	G	S	V	A	K	I	R	D	A	R	P	E	E	M	D	H
HuN4	HuN4	EF635006	G	H	G	T	A	K	I	R	D	T	R	P	E	E	L	N	H
	HuN4-F40	-	-	H	-	A	-	-	-	-	-	I	-	-	-	-	-	Y	Y
	HuN4-F60	-	-	H	-	A	-	-	-	-	-	I	-	-	-	-	-	Y	Y
	HuN4-F112	-	-	R	-	A	-	-	-	-	-	I	-	-	-	-	-	Y	Y
NT0801	NT0801	HQ315836	-	-	-	-	-	-	-	-	-	-	-	-	-	E	-	-	-
	NT0801-P10	KJ523894	-	-	-	-	-	-	-	-	-	-	-	-	-	E	-	-	-
	NT0801-P30	KJ523895	-	-	-	-	-	-	-	-	-	-	-	-	-	E	-	-	-

	NT0801-P50	KJ523896	-	-	-	-	-	-	-	-	-	-	-	-	-	E	-	-	-
	NT0801-P80	KJ523897	-	-	-	-	-	-	-	-	-	-	-	-	-	A	-	-	-
JXA1	JXA1	EF112445	-	-	-	-	-	-	-	-	-	T	-	P	-	-	L	-	-
	JXA1 P10	FJ548854	-	-	-	-	-	-	-	-	-	T	-	L	-	-	L	-	-
	JXA1 P15	FJ548855	-	-	-	-	-	-	-	-	-	T	-	P	-	-	L	-	-
	JXA1 P45	FJ548851	-	-	-	-	-	-	-	-	-	T	-	L	-	-	L	-	-
	JXA1 P70	FJ548852	-	-	-	-	-	-	-	-	-	I	-	P	-	-	L	-	-
	JXA1 P80	FJ548853	-	-	-	-	-	-	-	-	-	T	-	P	-	-	S	-	-
	JXA1 P100	KC422725	-	-	-	-	-	-	-	-	-	I	-	P	-	-	L	-	-
	JXA1 P110	KC422726	-	-	-	-	-	-	-	-	-	T	-	P	-	-	S	-	-
	JXA1 P120	KC422727	-	-	-	-	-	-	-	-	-	T	-	P	-	-	S	-	-
	JXA1-P130	KC422728	-	-	-	-	-	-	-	-	-	T	-	P	-	-	S	-	-
	JXA1-P140	KC422729	-	-	-	-	-	-	-	-	-	T	-	P	-	-	S	-	-
	JXA1-P150	KC422730	-	-	-	-	-	-	-	-	-	T	-	P	-	-	S	-	-
	JXA1-P160	KC422731	-	-	-	-	-	-	-	-	-	T	-	P	-	-	S	-	-
	JXA1-P170	JQ804986	-	-	-	-	-	-	-	-	-	T	-	P	-	-	S	-	-
JX143	JX143	EU708726	G	-	-	-	-	-	-	-	-	-	-	-	-	-	-	-	-
	JXM20	GQ499193	G	-	-	-	-	-	-	-	-	-	-	-	-	-	-	-	-
	JXM40	GQ499194	G	-	-	-	-	-	-	-	-	-	-	-	-	-	-	-	-
	JXM60	GQ499195	G	-	-	-	-	-	-	-	-	-	-	-	-	-	-	-	-
	JXM80	GQ499196	G	-	-	-	-	-	-	-	-	-	-	-	-	-	-	-	-
	JXM100	GQ475526	R	-	-	-	-	-	-	-	-	-	-	-	-	-	-	-	-
GD	GD	EU825724	-	-	-	-	-	-	-	-	-	-	-	-	-	-	-	-	-
	GD-P100	GU143913	-	-	-	-	-	-	-	-	-	-	-	-	-	-	-	-	-
GDQY1	GDQY1	JN387271	-	-	-	-	-	-	I	R	D	-	-	-	K	-	-	-	-
	GDQY1VP65	JN387272	-	-	-	-	-	-	T	G	G	-	-	-	K	-	-	-	-
	GDQY1VP80	JN387273	-	-	-	-	-	-	I	R	D	-	-	-	E	-	-	-	-
	GDQY1VP100	JN387274	-	-	-	-	-	-	I	R	G	-	-	-	E	-	-	-	-
BJ	BJ	EU825723	-	-	-	-	-	K	-	-	-	-	G	-	-	-	-	-	-
	BJ-F20	KP890337	-	-	-	-	-	K	-	-	-	-	G	-	-	-	-	-	-
	BJ-F40	KP890338	-	-	-	-	-	K	-	-	-	-	G	-	-	-	-	-	-

	BJ-F80	KP890340	-	-	-	-	-	K	-	-	-	-	R	-	-	-	-	-	-
	BJ-F100	KP890341	-	-	-	-	-	E	-	-	-	-	G	-	-	-	-	-	-
	BJ-F150	KP890342	-	-	-	-	-	K	-	-	-	-	R	-	-	-	-	-	-
TP	TP	EU864233	G	-	-	-	-	-	-	-	-	-	-	-	-	-	-	-	-
	TP_P60	GU232736	G	-	-	-	-	-	-	-	-	-	-	-	-	-	-	-	-
	TP_P90	GU232737	R	-	-	-	-	-	-	-	-	-	-	-	-	-	-	-	-
BB0907	BB0907	HQ315835	-	-	P	-	A	-	-	-	D	-	-	-	-	-	-	-	-
	BB0907-s34	KM453698	-	-	S	-	A	-	-	-	G	-	-	-	-	-	-	-	-
	BB0907-F44	KM453699	-	-	S	-	D	-	-	-	D	-	-	-	-	-	-	-	-
Series	Name	NO.	NSP2																
			218	215	222	228	254	297	298	310	326	330	330-417	390	398	400	411	415	417
	VR2332	EF536003	N	S	Q	I	A	E	A	Q	N	L	-	D	K	A	W	Q	D
HuN4	HuN4	EF635006	D	G	Q	I	A	E	A	L	D	L	-	D	K	A	C	Q	D
	HuN4-F40	-	-	R	-	-	-	-	-	S	-	-	-	-	-	-	W	-	N
	HuN4-F60	-	-	R	-	-	-	-	-	S	-	-	-	-	-	-	W	-	N
	HuN4-F112	-	-	R	-	-	-	-	-	S	-	-	-	-	-	-	W	-	-
NT0801	NT0801	HQ315836	N	R	-	-	-	-	-	S	-	P	-	-	-	V	R	-	-
	NT0801-P10	KJ523894	N	R	-	-	-	-	-	S	-	L	-	-	-	V	R	-	-
	NT0801-P30	KJ523895	N	R	-	-	-	-	-	S	-	L	-	-	-	V	R	-	-
	NT0801-P50	KJ523896	N	R	-	-	-	-	-	S	-	L	-	-	-	V	R	-	-
	NT0801-P80	KJ523897	N	R	-	-	-	-	-	S	-	L	-	-	-	V	R	-	-
JXA1	JXA1	EF112445	D	R	-	-	T	V	-	S	-	-	-	-	K	A	W	-	-
	JXA1 P10	FJ548854	D	R	-	-	T	V	-	S	-	-	-	-	K	T	W	-	-
	JXA1 P15	FJ548855	D	R	-	-	A	E	-	S	-	-	-	-	K	T	W	-	-
	JXA1 P45	FJ548851	D	R	-	-	A	E	-	S	-	-	-	-	K	T	W	-	-
	JXA1 P70	FJ548852	D	R	-	-	A	E	-	S	-	-	-	-	K	T	W	-	-
	JXA1 P80	FJ548853	D	R	-	-	A	E	-	S	-	-	-	-	K	T	W	-	-
	JXA1 P100	KC422725	D	R	-	-	A	E	-	S	-	-	-	-	K	T	W	-	-
	JXA1 P110	KC422726	D	R	-	-	A	E	-	S	-	-	-	-	K	T	W	-	-
	JXA1 P120	KC422727	D	R	-	-	A	E	-	S	-	-	-	-	Q	T	W	-	-
	JXA1-P130	KC422728	D	R	-	-	A	E	-	S	-	-	-	-	E	T	W	-	-

	JXA1-P140	KC422729	N	R	-	-	A	E	-	S	-	-	-	-	E	T	W	-	-
	JXA1-P150	KC422730	D	R	-	-	A	E	-	S	-	-	-	-	E	T	W	-	-
	JXA1-P160	KC422731	D	R	-	-	A	E	-	S	-	-	-	-	E	T	W	-	-
	JXA1-P170	JQ804986	D	R	-	-	A	E	-	S	-	-	-	-	E	T	W	-	-
JX143	JX143	EU708726	-	R	-	-	-	-	A	S	D	-	-	-	-	A	W	-	-
	JXM20	GQ499193	-	R	-	-	-	-	P	S	G	-	-	-	-	T	W	-	-
	JXM40	GQ499194	-	R	-	-	-	-	S	S	G	-	-	-	-	T	W	-	-
	JXM60	GQ499195	-	R	-	-	-	-	A	S	G	-	-	-	-	T	W	H	-
	JXM80	GQ499196	-	R	-	-	-	-	A	S	G	-	-	-	-	A	W	-	-
	JXM100	GQ475526	-	R	-	-	-	-	P	S	G	-	DEL	DEL	DEL	DEL	DEL	DEL	DEL
GD	GD	EU825724	-	R	-	-	-	-	-	S	-	-	-	D	-	-	W	-	-
	GD-P100	GU143913	-	R	-	-	-	-	-	S	-	-	-	N	-	-	W	-	-
GDQY1	GDQY1	JN387271	-	R	Q	I	-	-	-	S	-	-	-	-	K	-	W	-	-
	GDQY1VP65	JN387272	-	R	Q	I	-	-	-	S	-	-	-	-	K	-	W	-	-
	GDQY1VP80	JN387273	-	R	Q	I	-	-	-	S	-	-	-	-	E	-	W	-	-
	GDQY1VP100	JN387274	-	R	R	T	-	-	-	S	-	-	-	-	E	-	W	-	-
BJ	BJ	EU825723	-	I	-	-	-	-	-	S	G	-	-	-	K	-	W	-	-
	BJ-F20	KP890337	-	I	-	-	-	-	-	S	G	-	-	-	K	-	W	-	-
	BJ-F40	KP890338	-	I	-	-	-	-	-	S	G	-	-	-	E	-	W	-	-
	BJ-F80	KP890340	-	I	-	-	-	-	-	S	G	-	-	-	E	-	W	-	-
	BJ-F100	KP890341	-	I	-	-	-	-	-	S	G	-	-	-	E	-	W	-	-
	BJ-F150	KP890342	-	I	-	-	-	-	-	S	G	-	-	-	E	-	W	-	-
TP	TP	EU864233	-	R	-	-	-	-	-	S	-	-	-	-	K	-	W	-	-
	TP_P60	GU232736	-	R	-	-	-	-	-	S	-	-	-	-	K	-	W	-	-
	TP_P90	GU232737	-	R	-	-	-	-	-	S	-	-	-	-	E	-	W	-	-
BB0907	BB0907	HQ315835	-	-	-	-	-	-	-	S	-	-	-	-	K	-	R	-	-
	BB0907-s34	KM453698	-	-	-	-	-	-	-	S	-	-	-	-	K	-	R	-	-
	BB0907-F44	KM453699	-	-	-	-	-	-	-	S	-	-	-	-	K	-	W	-	-
Series	Name	NO.	NSP2																
			432	451	464	465	466	471	483	487	497	465-499	500	521	533	568	575	577	580
	VR2332	EF536003	P	R	D	C	G	L	A	P	A	-	S	E	S	Q	S	S	T

HuN4	HuN4	EF635006	P	D	D	C	G	M	T	P	V	-	M	G	T	Q	S	S	T
	HuN4-F40	-	-	G	-	-	-	-	-	-	M	-	-	-	-	-	P	-	-
	HuN4-F60	-	-	G	-	-	-	-	-	-	M	-	-	-	-	-	P	-	-
	HuN4-F112	-	-	G	-	-	-	-	-	-	M	-	-	-	-	-	P	-	-
NT0801	NT0801	HQ315836	-	-	D	-	-	-	-	-	M	-	-	-	S	Q	-	-	T
	NT0801-P10	KJ523894	-	-	D	-	-	-	-	-	M	-	-	-	S	Q	-	-	T
	NT0801-P30	KJ523895	-	-	D	-	-	-	-	-	M	-	-	-	S	Q	-	-	T
	NT0801-P50	KJ523896	-	-	G	-	-	-	-	-	M	-	-	-	S	R	-	-	A
	NT0801-P80	KJ523897	-	-	G	-	-	-	-	-	M	-	-	-	S	R	-	-	A
JXA1	JXA1	EF112445	-	G	-	-	G	M	-	-	M	-	-	G	-	-	-	-	-
	JXA1 P10	FJ548854	-	G	-	-	G	M	-	-	M	-	-	G	-	-	-	-	-
	JXA1 P15	FJ548855	-	G	-	-	G	M	-	-	M	-	-	G	-	-	-	-	-
	JXA1 P45	FJ548851	-	G	-	-	G	M	-	-	M	-	-	G	-	-	-	-	-
	JXA1 P70	FJ548852	-	G	-	-	S	M	-	-	M	-	-	G	-	-	-	-	-
	JXA1 P80	FJ548853	-	G	-	-	G	M	-	-	M	-	-	G	-	-	-	-	-
	JXA1 P100	KC422725	-	G	-	-	G	M	-	-	M	-	-	G	-	-	-	-	-
	JXA1 P110	KC422726	-	G	-	-	G	M	-	-	M	-	-	G	-	-	-	-	-
	JXA1 P120	KC422727	-	G	-	-	G	M	-	-	M	-	-	G	-	-	-	-	-
	JXA1-P130	KC422728	-	G	-	-	G	M	-	-	M	-	-	G	-	-	-	-	-
	JXA1-P140	KC422729	-	G	-	-	G	M	-	-	M	-	-	G	-	-	-	-	-
	JXA1-P150	KC422730	-	G	-	-	G	M	-	-	M	-	-	G	-	-	-	-	-
	JXA1-P160	KC422731	-	G	-	-	G	T	-	-	M	-	-	E	-	-	-	-	-
	JXA1-P170	JQ804986	-	G	-	-	G	M	-	-	M	-	-	E	-	-	-	-	-
JX143	JX143	EU708726	P	-	C	-	-	-	-	-	M	-	-	-	T	S	-	-	-
	JXM20	GQ499193	P	-	C	-	-	-	-	-	M	-	-	-	T	S	-	-	-
	JXM40	GQ499194	P	-	C	-	-	-	-	-	M	-	-	-	I	S	-	-	-
	JXM60	GQ499195	S	-	R	-	-	-	-	-	M	-	-	-	T	S	-	-	-
	JXM80	GQ499196	P	-	C	-	-	-	-	-	M	-	-	-	I	S	-	-	-
	JXM100	GQ475526	P	-	C	-	-	-	-	-	M	-	-	-	I	P	-	-	-
GD	GD	EU825724	-	G	-	-	-	-	-	-	M	-	-	-	-	-	-	-	-
	GD-P100	GU143913	-	G	-	-	-	-	-	-	M	-	-	-	-	-	-	-	-

GDQY1	GDQY1	JN387271	-	G	-	-	-	-	-	-	M	-	M	-	-	-	-	-	-
	GDQY1VP65	JN387272	-	G	-	-	-	-	-	-	M	-	M	-	-	-	-	-	-
	GDQY1VP80	JN387273	-	G	-	DEL	DEL	-	-	-	M	DEL	L	-	-	-	-	-	-
	GDQY1VP100	JN387274	-	G	-	DEL	DEL	-	-	-	M	DEL	M	-	-	-	-	-	-
BJ	BJ	EU825723	-	-	-	-	-	-	-	L	M	-	-	-	-	-	-	-	-
	BJ-F20	KP890337	-	-	-	-	-	-	-	L	M	-	-	-	-	-	-	-	-
	BJ-F40	KP890338	-	-	-	-	-	-	-	L	M	-	-	-	-	-	-	-	-
	BJ-F80	KP890340	-	-	-	-	-	-	-	L	M	-	-	-	-	-	-	-	-
	BJ-F100	KP890341	-	-	-	-	-	-	-	L	M	-	-	-	-	-	-	-	-
	BJ-F150	KP890342	-	-	-	-	-	-	-	L	M	-	-	-	-	-	-	-	-
TP	TP	EU864233	-	G	-	-	-	-	T	-	M	-	-	-	-	-	-	S	-
	TP_P60	GU232736	-	G	-	-	-	-	T	-	M	-	-	-	-	-	-	P	-
	TP_P90	GU232737	-	G	-	-	-	-	I	-	M	-	-	-	-	-	-	P	-
BB0907	BB0907	HQ315835	-	-	-	-	-	-	-	P	M	-	-	-	-	-	-	-	-
	BB0907-s34	KM453698	-	-	-	-	-	-	-	S	M	-	-	-	-	-	-	-	-
	BB0907-F44	KM453699	-	-	-	-	-	-	-	P	M	-	-	-	-	-	-	-	-
Series	Name	NO.	NSP2																
			581	582	590	599	605	613	614	627	645	675	678	697	700	711	784	793	802
	VR2332	EF536003	E	Y	P	E	E	M	S	S	D	D	A	N	V	L	D	I	T
HuN4	HuN4	EF635006	E	Y	S	G	E	I	L	S	D	S	D	N	A	F	C	M	I
	HuN4-F40	-	-	-	-	-	-	-	-	P	-	G	A	-	-	-	S	-	M
	HuN4-F60	-	-	-	-	-	-	-	-	P	-	G	A	-	-	-	S	-	M
	HuN4-F112	-	-	-	-	-	-	-	-	P	-	G	A	-	-	-	S	-	M
NT0801	NT0801	HQ315836	-	-	-	V	-	-	-	-	-	G	A	-	-	L	-	I	V
	NT0801-P10	KJ523894	-	-	-	V	-	-	-	-	-	G	A	-	-	L	-	I	V
	NT0801-P30	KJ523895	-	-	-	V	-	-	-	-	-	G	A	-	-	L	-	I	V
	NT0801-P50	KJ523896	-	-	-	V	-	-	-	-	-	G	A	-	-	L	-	I	V
	NT0801-P80	KJ523897	-	-	-	V	-	-	-	-	-	G	A	-	-	L	-	I	V
JXA1	JXA1	EF112445	E	-	-	G	E	-	-	-	-	G	A	-	-	-	-	-	M
	JXA1 P10	FJ548854	E	-	-	R	G	-	-	-	-	G	A	-	-	-	-	-	M
	JXA1 P15	FJ548855	E	-	-	R	G	-	-	-	-	G	A	-	-	-	-	-	M

	JXA1 P45	FJ548851	E	-	-	R	G	-	-	-	-	G	A	-	-	-	-	-	M
	JXA1 P70	FJ548852	E	-	-	R	G	-	-	-	-	G	A	-	-	-	-	-	M
	JXA1 P80	FJ548853	E	-	-	R	G	-	-	-	-	G	A	-	-	-	-	-	M
	JXA1 P100	KC422725	E	-	-	R	G	-	-	-	-	G	A	-	-	-	-	-	M
	JXA1 P110	KC422726	E	-	-	R	G	-	-	-	-	G	A	-	-	-	-	-	M
	JXA1 P120	KC422727	E	-	-	R	G	-	-	-	-	G	A	-	-	-	-	-	M
	JXA1-P130	KC422728	E	-	-	R	G	-	-	-	-	G	A	-	-	-	-	-	M
	JXA1-P140	KC422729	E	-	-	R	G	-	-	-	-	G	A	-	-	-	-	-	M
	JXA1-P150	KC422730	E	-	-	R	G	-	-	-	-	G	A	-	-	-	-	-	M
	JXA1-P160	KC422731	G	-	-	R	G	-	-	-	-	G	A	-	-	-	-	-	M
	JXA1-P170	JQ804986	E	-	-	R	G	-	-	-	-	G	A	-	-	-	-	-	M
JX143	JX143	EU708726	-	Y	-	-	-	-	-	-	-	-	A	-	-	-	-	-	-
	JXM20	GQ499193	-	Y	-	-	-	-	-	-	-	-	A	-	-	-	-	-	-
	JXM40	GQ499194	-	Y	-	-	-	-	-	-	-	-	A	-	-	-	-	-	-
	JXM60	GQ499195	-	Y	-	-	-	-	-	-	-	-	A	-	-	-	-	-	-
	JXM80	GQ499196	-	Y	-	-	-	-	-	-	-	-	A	-	-	-	-	-	-
	JXM100	GQ475526	-	H	-	-	-	-	-	-	-	-	A	-	-	-	-	-	-
GD	GD	EU825724	-	-	-	-	-	-	-	-	-	G	A	-	-	F	-	-	M
	GD-P100	GU143913	-	-	-	-	-	-	-	-	-	G	A	-	-	L	-	-	M
GDQY1	GDQY1	JN387271	E	-	-	-	-	-	-	-	D	G	A	-	A	-	-	-	M
	GDQY1VP65	JN387272	G	-	-	-	-	-	-	-	N	G	A	-	A	-	-	-	M
	GDQY1VP80	JN387273	E	-	-	-	-	-	-	-	N	G	A	-	T	-	-	-	M
	GDQY1VP100	JN387274	G	-	-	-	-	-	-	-	N	G	A	-	A	-	-	-	M
BJ	BJ	EU825723	-	-	-	-	-	V	-	-	-	-	A	-	-	-	-	-	-
	BJ-F20	KP890337	-	-	-	-	-	V	-	-	-	-	A	-	-	-	-	-	-
	BJ-F40	KP890338	-	-	-	-	-	V	-	-	-	-	A	-	-	-	-	-	-
	BJ-F80	KP890340	-	-	-	-	-	V	-	-	-	-	A	-	-	-	-	-	-
	BJ-F100	KP890341	-	-	-	-	-	V	-	-	-	-	A	-	-	-	-	-	-
	BJ-F150	KP890342	-	-	-	-	-	V	-	-	-	-	A	-	-	-	-	-	-
TP	TP	EU864233	-	-	S	-	-	I	-	-	-	G	A	N	-	F	-	M	M
	TP_P60	GU232736	-	-	S	-	-	I	-	-	-	G	A	D	-	F	-	M	M

	TP_P90	GU232737	-	-	P	-	-	T	-	-	-	G	A	D	-	S	-	T	M
BB0907	BB0907	HQ315835	-	K	-	-	-	-	L	-	-	-	A	-	-	-	-	-	-
	BB0907-s34	KM453698	-	K	-	-	-	-	L	-	-	-	A	-	-	-	-	-	-
	BB0907-F44	KM453699	-	K	-	-	-	-	P	-	-	-	A	-	-	-	-	-	-
Series	Name	NO.	NSP2							NSP3									NSP4
			803	809	814	815	1040	1099	1177	6	13	86	95	96	133	164	175	176	60
	VR2332	EF536003	S	T	S	F	S	A	V	M	C	V	M	A	C	V	V	A	D
HuN4	HuN4	EF635006	I	T	S	F	S	A	T	I	C	V	V	A	W	V	I	A	D
	HuN4-F40	-	-	-	-	-	-	-	-	-	-	-	M	A	-	-	-	-	-
	HuN4-F60	-	-	-	-	-	-	-	-	-	-	-	M	V	-	-	-	-	-
	HuN4-F112	-	-	-	-	-	-	-	-	-	-	-	M	V	-	-	-	-	-
NT0801	NT0801	HQ315836	M	-	-	-	-	-	I	F	-	-	M	-	-	-	-	-	-
	NT0801-P10	KJ523894	M	-	-	-	-	-	I	F	-	-	M	-	-	-	-	-	-
	NT0801-P30	KJ523895	M	-	-	-	-	-	I	F	-	-	M	-	-	-	-	-	-
	NT0801-P50	KJ523896	M	-	-	-	-	-	I	F	-	-	M	-	-	-	-	-	-
	NT0801-P80	KJ523897	M	-	-	-	-	-	I	F	-	-	M	-	-	-	-	-	-
JXA1	JXA1	EF112445	I	-	-	-	S	T	-	-	R	-	M	-	-	-	I	-	-
	JXA1 P10	FJ548854	I	-	-	-	L	A	-	-	C	-	M	-	-	-	I	-	-
	JXA1 P15	FJ548855	I	-	-	-	L	A	-	-	C	-	M	-	-	-	I	-	-
	JXA1 P45	FJ548851	I	-	-	-	L	A	-	-	C	-	M	-	-	-	I	-	-
	JXA1 P70	FJ548852	I	-	-	-	L	T	-	-	C	-	M	-	-	-	I	-	-
	JXA1 P80	FJ548853	M	-	-	-	L	T	-	-	C	-	M	-	-	-	I	-	-
	JXA1 P100	KC422725	M	-	-	-	L	T	-	-	C	-	M	-	-	-	I	-	-
	JXA1 P110	KC422726	M	-	-	-	L	T	-	-	C	-	M	-	-	-	T	-	-
	JXA1 P120	KC422727	I	-	-	-	L	T	-	-	C	-	M	-	-	-	T	-	-
	JXA1-P130	KC422728	M	-	-	-	L	T	-	-	C	-	M	-	-	-	T	-	-
	JXA1-P140	KC422729	I	-	-	-	L	T	-	-	C	-	M	-	-	-	T	-	-
	JXA1-P150	KC422730	M	-	-	-	L	T	-	-	C	-	M	-	-	-	T	-	-
	JXA1-P160	KC422731	M	-	-	-	L	T	-	-	C	-	M	-	-	-	T	-	-
	JXA1-P170	JQ804986	I	-	-	-	L	T	-	-	C	-	M	-	-	-	T	-	-
JX143	JX143	EU708726	-	-	-	-	-	-	-	-	-	-	M	-	C	I	V	-	D

	JXM20	GQ499193	-	-	-	-	-	-	-	-	-	-	M	-	W	V	I	-	A
	JXM40	GQ499194	-	-	-	-	-	-	-	-	-	-	M	-	W	V	I	-	A
	JXM60	GQ499195	-	-	-	-	-	-	-	-	-	-	M	-	W	V	I	-	A
	JXM80	GQ499196	-	-	-	-	-	-	-	-	-	-	M	-	W	V	I	-	A
	JXM100	GQ475526	-	-	-	-	-	-	-	-	-	-	M	-	W	V	I	-	A
GD	GD	EU825724	-	-	-	-	-	-	-	-	-	V	M	-	-	-	-	-	-
	GD-P100	GU143913	-	-	-	-	-	-	-	-	-	G	M	-	-	-	-	-	-
GDQY1	GDQY1	JN387271	-	-	-	-	-	-	-	-	-	-	M	A	-	-	-	-	-
	GDQY1VP65	JN387272	-	-	-	-	-	-	-	-	-	-	M	A	-	-	-	-	-
	GDQY1VP80	JN387273	-	-	-	-	-	-	-	-	-	-	M	A	-	-	-	-	-
	GDQY1VP100	JN387274	-	-	-	-	-	-	-	-	-	-	M	V	-	-	-	-	-
BJ	BJ	EU825723	-	T	-	-	-	-	-	I	-	-	M	-	-	-	-	A	-
	BJ-F20	KP890337	-	T	-	-	-	-	-	I	-	-	M	-	-	-	-	A	-
	BJ-F40	KP890338	-	T	-	-	-	-	-	I	-	-	M	-	-	-	-	A	-
	BJ-F80	KP890340	-	A	-	-	-	-	-	T	-	-	M	-	-	-	-	A	-
	BJ-F100	KP890341	-	A	-	-	-	-	-	T	-	-	M	-	-	-	-	T	-
	BJ-F150	KP890342	-	A	-	-	-	-	-	T	-	-	M	-	-	-	-	A	-
TP	TP	EU864233	-	-	-	-	-	-	T	-	-	-	M	-	-	-	I	-	-
	TP_P60	GU232736	-	-	-	-	-	-	A	-	-	-	M	-	-	-	I	-	-
	TP_P90	GU232737	-	-	-	-	-	-	T	-	-	-	M	-	-	-	T	-	-
BB0907	BB0907	HQ315835	-	-	S	F	-	-	-	-	-	-	M	-	-	-	-	-	-
	BB0907-s34	KM453698	-	-	L	F	-	-	-	-	-	-	M	-	-	-	-	-	-
	BB0907-F44	KM453699	-	-	S	S	-	-	-	-	-	-	M	-	-	-	-	-	-
Series	Name	NO.	NSP4								NSP5			NSP7					
			81	92	151	155	163	175	184	199	7	85	120	51	52	76	96	104	117
	VR2332	EF536003	Q	Y	F	A	S	D	K	A	Q	S	M	V	E	V	S	S	L
HuN4	HuN4	EF635006	Q	Y	F	K	C	D	K	A	Q	S	I	I	E	V	S	S	L
	HuN4-F40	-	-	-	-	-	S	-	-	A	-	-	M	I	-	-	-	-	-
	HuN4-F60	-	-	-	-	-	S	-	-	A	-	-	M	I	-	-	-	-	-
	HuN4-F112	-	-	-	-	-	S	-	-	V	-	-	M	V	-	-	-	-	-
NT0801	NT0801	HQ315836	-	-	-	T	S	-	-	-	-	-	M	-	-	-	-	-	-

	NT0801-P10	KJ523894	-	-	-	T	S	-	-	-	-	-	M	-	-	-	-	-	-
	NT0801-P30	KJ523895	-	-	-	T	S	-	-	-	-	-	M	-	-	-	-	-	-
	NT0801-P50	KJ523896	-	-	-	T	S	-	-	-	-	-	M	-	-	-	-	-	-
	NT0801-P80	KJ523897	-	-	-	T	S	-	-	-	-	-	M	-	-	-	-	-	-
JXA1	JXA1	EF112445	-	-	-	K	S	-	K	-	-	-	M	-	-	-	-	-	-
	JXA1 P10	FJ548854	-	-	-	K	S	-	K	-	-	-	M	-	-	-	-	-	-
	JXA1 P15	FJ548855	-	-	-	K	S	-	K	-	-	-	M	-	-	-	-	-	-
	JXA1 P45	FJ548851	-	-	-	K	S	-	K	-	-	-	M	-	-	-	-	-	-
	JXA1 P70	FJ548852	-	-	-	K	S	-	K	-	-	-	M	-	-	-	-	-	-
	JXA1 P80	FJ548853	-	-	-	K	S	-	K	-	-	-	M	-	-	-	-	-	-
	JXA1 P100	KC422725	-	-	-	K	S	-	K	-	-	-	M	-	-	-	-	-	-
	JXA1 P110	KC422726	-	-	-	K	S	-	K	-	-	-	M	-	-	-	-	-	-
	JXA1 P120	KC422727	-	-	-	K	S	-	K	-	-	-	M	-	-	-	-	-	-
	JXA1-P130	KC422728	-	-	-	K	S	-	E	-	-	-	M	-	-	-	-	-	-
	JXA1-P140	KC422729	-	-	-	K	S	-	K	-	-	-	M	-	-	-	-	-	-
	JXA1-P150	KC422730	-	-	-	K	S	-	K	-	-	-	M	-	-	-	-	-	-
	JXA1-P160	KC422731	-	-	-	Q	S	-	K	-	-	-	M	-	-	-	-	-	-
	JXA1-P170	JQ804986	-	-	-	K	S	-	K	-	-	-	M	-	-	-	-	-	-
JX143	JX143	EU708726	-	-	-	-	S	-	-	-	-	-	M	-	E	-	-	S	-
	JXM20	GQ499193	-	-	-	-	S	-	-	-	-	-	M	-	E	-	-	S	-
	JXM40	GQ499194	-	-	-	-	S	-	-	-	-	-	M	-	G	-	-	S	-
	JXM60	GQ499195	-	-	-	-	S	-	-	-	-	-	M	-	E	-	-	S	-
	JXM80	GQ499196	-	-	-	-	S	-	-	-	-	-	M	-	E	-	-	S	-
	JXM100	GQ475526	-	-	-	-	S	-	-	-	-	-	M	-	E	-	-	G	-
GD	GD	EU825724	Q	Y	-	-	S	-	-	-	-	S	M	-	-	-	-	-	-
	GD-P100	GU143913	R	H	-	-	S	-	-	-	-	C	M	-	-	-	-	-	-
GDQY1	GDQY1	JN387271	-	-	-	-	S	-	K	-	K	-	M	-	-	-	-	-	-
	GDQY1VP65	JN387272	-	-	-	-	S	-	E	-	Q	-	M	-	-	-	-	-	-
	GDQY1VP80	JN387273	-	-	-	-	S	-	E	-	Q	-	M	-	-	-	-	-	-
	GDQY1VP100	JN387274	-	-	-	-	S	-	E	-	Q	-	M	-	-	-	-	-	-
BJ	BJ	EU825723	-	-	F	-	S	-	-	-	-	-	M	-	-	V	S	-	L

	BJ-F20	KP890337	-	-	F	-	S	-	-	-	-	-	M	-	-	V	S	-	L
	BJ-F40	KP890338	-	-	F	-	S	-	-	-	-	-	M	-	-	A	S	-	L
	BJ-F80	KP890340	-	-	F	-	S	-	-	-	-	-	M	-	-	V	S	-	L
	BJ-F100	KP890341	-	-	L	-	S	-	-	-	-	-	M	-	-	V	S	-	L
	BJ-F150	KP890342	-	-	F	-	S	-	-	-	-	-	M	-	-	V	N	-	F
TP	TP	EU864233	-	Y	-	-	S	D	-	-	-	-	M	-	-	-	-	-	-
	TP_P60	GU232736	-	H	-	-	S	D	-	-	-	-	M	-	-	-	-	-	-
	TP_P90	GU232737	-	H	-	-	S	E	-	-	-	-	M	-	-	-	-	-	-
BB0907	BB0907	HQ315835	-	Y	-	-	S	-	-	-	-	-	M	-	-	-	-	-	-
	BB0907-s34	KM453698	-	H	-	-	S	-	-	-	-	-	M	-	-	-	-	-	-
	BB0907-F44	KM453699	-	Y	-	-	S	-	-	-	-	-	M	-	-	-	-	-	-
Series	Name	NO.	NSP7						NSP9										
			146	150	162	166	200	205	4	19	67	99	120	150	178	214	225	235	239
	VR2332	EF536003	K	N	N	R	N	W	S	T	L	G	A	E	A	S	C	E	L
HuN4	HuN4	EF635006	K	N	N	R	I	W	S	T	L	G	A	E	A	S	C	E	L
	HuN4-F40	-	-	-	-	-	-	-	-	A	-	-	A	-	-	P	-	-	-
	HuN4-F60	-	-	-	-	-	-	-	-	A	-	-	A	-	-	P	-	-	-
	HuN4-F112	-	-	-	-	-	-	-	-	A	-	-	V	-	-	P	-	-	-
NT0801	NT0801	HQ315836	-	-	-	-	-	-	-	-	-	-	-	-	-	-	-	-	-
	NT0801-P10	KJ523894	-	-	-	-	-	-	-	-	-	-	-	-	-	-	-	-	-
	NT0801-P30	KJ523895	-	-	-	-	-	-	-	-	-	-	-	-	-	-	-	-	-
	NT0801-P50	KJ523896	-	-	-	-	-	-	-	-	-	-	-	-	-	-	-	-	-
	NT0801-P80	KJ523897	-	-	-	-	-	-	-	-	-	-	-	-	-	-	-	-	-
JXA1	JXA1	EF112445	-	-	N	-	I	-	-	-	-	-	-	-	-	-	-	-	-
	JXA1 P10	FJ548854	-	-	S	-	I	-	-	-	-	-	-	-	-	-	-	-	-
	JXA1 P15	FJ548855	-	-	S	-	I	-	-	-	-	-	-	-	-	-	-	-	-
	JXA1 P45	FJ548851	-	-	S	-	I	-	-	-	-	-	-	-	-	-	-	-	-
	JXA1 P70	FJ548852	-	-	S	-	I	-	-	-	-	-	-	-	-	-	-	-	-
	JXA1 P80	FJ548853	-	-	S	-	I	-	-	-	-	-	-	-	-	-	-	-	-
	JXA1 P100	KC422725	-	-	S	-	I	-	-	-	-	-	-	-	-	-	-	-	-
	JXA1 P110	KC422726	-	-	S	-	I	-	-	-	-	-	-	-	-	-	-	-	-

	JXA1 P120	KC422727	-	-	S	-	I	-	-	-	-	-	-	-	-	-	-	-	-
	JXA1-P130	KC422728	-	-	S	-	I	-	-	-	-	-	-	-	-	-	-	-	-
	JXA1-P140	KC422729	-	-	S	-	I	-	-	-	-	-	-	-	-	-	-	-	-
	JXA1-P150	KC422730	-	-	S	-	I	-	-	-	-	-	-	-	-	-	-	-	-
	JXA1-P160	KC422731	-	-	S	-	T	-	-	-	-	-	-	-	-	-	-	-	-
	JXA1-P170	JQ804986	-	-	S	-	I	-	-	-	-	-	-	-	-	-	-	-	-
JX143	JX143	EU708726	-	N	N	R	-	-	-	-	L	G	-	E	-	-	-	-	-
	JXM20	GQ499193	-	N	N	R	-	-	-	-	F	G	-	G	-	-	-	-	-
	JXM40	GQ499194	-	N	N	G	-	-	-	-	L	G	-	E	-	-	-	-	-
	JXM60	GQ499195	-	S	K	R	-	-	-	-	L	G	-	E	-	-	-	-	-
	JXM80	GQ499196	-	N	N	R	-	-	-	-	L	R	-	E	-	-	-	-	-
	JXM100	GQ475526	-	S	N	R	-	-	-	-	L	G	-	E	-	-	-	-	-
GD	GD	EU825724	-	-	-	-	-	W	-	-	-	-	-	-	-	-	-	-	-
	GD-P100	GU143913	-	-	-	-	-	R	-	-	-	-	-	-	-	-	-	-	-
GDQY1	GDQY1	JN387271	K	-	-	-	-	-	-	-	-	-	-	-	A	-	C	E	L
	GDQY1VP65	JN387272	K	-	-	-	-	-	-	-	-	-	-	-	A	-	C	E	L
	GDQY1VP80	JN387273	N	-	-	-	-	-	-	-	-	-	-	-	A	-	F	K	I
	GDQY1VP100	JN387274	N	-	-	-	-	-	-	-	-	-	-	-	S	-	C	K	L
BJ	BJ	EU825723	-	-	-	-	-	-	-	-	-	-	-	-	-	-	-	-	-
	BJ-F20	KP890337	-	-	-	-	-	-	-	-	-	-	-	-	-	-	-	-	-
	BJ-F40	KP890338	-	-	-	-	-	-	-	-	-	-	-	-	-	-	-	-	-
	BJ-F80	KP890340	-	-	-	-	-	-	-	-	-	-	-	-	-	-	-	-	-
	BJ-F100	KP890341	-	-	-	-	-	-	-	-	-	-	-	-	-	-	-	-	-
	BJ-F150	KP890342	-	-	-	-	-	-	-	-	-	-	-	-	-	-	-	-	-
TP	TP	EU864233	-	-	-	-	-	-	-	-	-	-	-	-	-	T	-	-	-
	TP_P60	GU232736	-	-	-	-	-	-	-	-	-	-	-	-	-	T	-	-	-
	TP_P90	GU232737	-	-	-	-	-	-	-	-	-	-	-	-	-	T	-	-	-
BB0907	BB0907	HQ315835	-	-	-	-	-	-	S	-	-	-	-	-	-	-	-	-	-
	BB0907-s34	KM453698	-	-	-	-	-	-	G	-	-	-	-	-	-	-	-	-	-
	BB0907-F44	KM453699	-	-	-	-	-	-	G	-	-	-	-	-	-	-	-	-	-
Series	Name	NO.	NSP9							NSP10									NSP11

			254	274	362	420	411	502	554	6	59	69	73	114	204	275	366	393	52
	VR2332	EF536003	L	P	M	E	V	I	I	V	K	E	Y	D	R	T	H	A	A
HuN4	HuN4	EF635006	L	P	M	E	V	I	I	M	K	E	Y	D	R	T	H	A	A
	HuN4-F40	-	-	-	-	-	-	I	-	-	K	E	-	-	-	-	-	-	-
	HuN4-F60	-	-	-	-	-	-	I	-	-	K	G	-	-	-	-	-	-	-
	HuN4-F112	-	-	-	-	-	-	L	-	-	R	G	-	-	-	-	-	-	-
NT0801	NT0801	HQ315836	-	-	-	-	-	-	-	-	-	-	-	-	-	-	-	-	-
	NT0801-P10	KJ523894	-	-	-	-	-	-	-	-	-	-	-	-	-	-	-	-	-
	NT0801-P30	KJ523895	-	-	-	-	-	-	-	-	-	-	-	-	-	-	-	-	-
	NT0801-P50	KJ523896	-	-	-	-	-	-	-	-	-	-	-	-	-	-	-	-	-
	NT0801-P80	KJ523897	-	-	-	-	-	-	-	-	-	-	-	-	-	-	-	-	-
JXA1	JXA1	EF112445	-	-	I	E	-	-	-	-	-	-	-	-	R	-	-	A	A
	JXA1 P10	FJ548854	-	-	M	E	-	-	-	-	-	-	-	-	H	-	-	T	A
	JXA1 P15	FJ548855	-	-	M	G	-	-	-	-	-	-	-	-	H	-	-	T	A
	JXA1 P45	FJ548851	-	-	M	G	-	-	-	-	-	-	-	-	H	-	-	T	V
	JXA1 P70	FJ548852	-	-	M	G	-	-	-	-	-	-	-	-	H	-	-	T	V
	JXA1 P80	FJ548853	-	-	M	G	-	-	-	-	-	-	-	-	H	-	-	T	V
	JXA1 P100	KC422725	-	-	M	G	-	-	-	-	-	-	-	-	H	-	-	T	V
	JXA1 P110	KC422726	-	-	M	G	-	-	-	-	-	-	-	-	H	-	-	T	A
	JXA1 P120	KC422727	-	-	M	G	-	-	-	-	-	-	-	-	H	-	-	T	A
	JXA1-P130	KC422728	-	-	M	G	-	-	-	-	-	-	-	-	H	-	-	T	A
	JXA1-P140	KC422729	-	-	M	G	-	-	-	-	-	-	-	-	H	-	-	T	A
	JXA1-P150	KC422730	-	-	M	G	-	-	-	-	-	-	-	-	H	-	-	T	A
	JXA1-P160	KC422731	-	-	M	G	-	-	-	-	-	-	-	-	H	-	-	T	A
	JXA1-P170	JQ804986	-	-	M	G	-	-	-	-	-	-	-	-	H	-	-	T	A
JX143	JX143	EU708726	-	-	-	-	V	-	-	-	-	-	Y	D	-	T	H	-	-
	JXM20	GQ499193	-	-	-	-	V	-	-	-	-	-	Y	D	-	T	H	-	-
	JXM40	GQ499194	-	-	-	-	A	-	-	-	-	-	C	D	-	A	L	-	-
	JXM60	GQ499195	-	-	-	-	V	-	-	-	-	-	Y	D	-	T	H	-	-
	JXM80	GQ499196	-	-	-	-	V	-	-	-	-	-	Y	D	-	T	H	-	-
	JXM100	GQ475526	-	-	-	-	V	-	-	-	-	-	Y	N	-	A	H	-	-

GD	GD	EU825724	-	-	-	-	-	-	-	-	-	-	-	-	-	-	-	-	-
	GD-P100	GU143913	-	-	-	-	-	-	-	-	-	-	-	-	-	-	-	-	-
GDQY1	GDQY1	JN387271	-	P	-	-	-	-	I	-	-	-	-	-	-	-	-	-	-
	GDQY1VP65	JN387272	-	S	-	-	-	-	I	-	-	-	-	-	-	-	-	-	-
	GDQY1VP80	JN387273	-	S	-	-	-	-	M	-	-	-	-	-	-	-	-	-	-
	GDQY1VP100	JN387274	-	S	-	-	-	-	M	-	-	-	-	-	-	-	-	-	-
BJ	BJ	EU825723	L	-	-	-	-	-	-	M	-	E	-	-	-	-	-	-	-
	BJ-F20	KP890337	L	-	-	-	-	-	-	M	-	E	-	-	-	-	-	-	-
	BJ-F40	KP890338	L	-	-	-	-	-	-	M	-	E	-	-	-	-	-	-	-
	BJ-F80	KP890340	L	-	-	-	-	-	-	M	-	E	-	-	-	-	-	-	-
	BJ-F100	KP890341	F	-	-	-	-	-	-	I	-	G	-	-	-	-	-	-	-
	BJ-F150	KP890342	F	-	-	-	-	-	-	I	-	G	-	-	-	-	-	-	-
TP	TP	EU864233	-	P	-	-	-	-	-	-	-	-	-	-	-	-	-	A	-
	TP_P60	GU232736	-	S	-	-	-	-	-	-	-	-	-	-	-	-	-	T	-
	TP_P90	GU232737	-	S	-	-	-	-	-	-	-	-	-	-	-	-	-	T	-
BB0907	BB0907	HQ315835	-	P	-	-	-	-	-	-	-	-	-	-	-	-	-	-	-
	BB0907-s34	KM453698	-	S	-	-	-	-	-	-	-	-	-	-	-	-	-	-	-
	BB0907-F44	KM453699	-	P	-	-	-	-	-	-	-	-	-	-	-	-	-	-	-
Series	Name	NO.	NSP11													NSP12			
			89	97	103	125	145	158	175	191	195	197	202	215	216	43	118	121	134
	VR2332	EF536003	K	V	F	A	H	E	L	H	Q	K	M	D	K	T	D	R	T
HuN4	HuN4	EF635006	K	V	F	E	H	E	V	H	P	K	T	G	R	T	D	R	T
	HuN4-F40	-	-	M	F	-	-	-	-	Y	Q	R	M	D	K	-	-	-	I
	HuN4-F60	-	-	M	L	-	-	-	-	Y	Q	R	M	D	K	-	-	-	I
	HuN4-F112	-	-	M	F	-	-	-	-	Y	Q	R	M	D	K	-	-	-	I
NT0801	NT0801	HQ315836	-	-	-	-	-	-	-	-	Q	R	M	-	K	-	-	-	-
	NT0801-P10	KJ523894	-	-	-	-	-	-	-	-	Q	R	M	-	K	-	-	-	-
	NT0801-P30	KJ523895	-	-	-	-	-	-	-	-	Q	R	M	-	K	-	-	-	-
	NT0801-P50	KJ523896	-	-	-	-	-	-	-	-	Q	R	M	-	K	-	-	-	-
	NT0801-P80	KJ523897	-	-	-	-	-	-	-	-	Q	R	M	-	K	-	-	-	-
JXA1	JXA1	EF112445	-	M	-	-	-	-	-	-	Q	R	M	D	K	-	-	-	-

	JXA1 P10	FJ548854	-	V	-	-	-	-	-	-	Q	R	M	D	K	-	-	-	-
	JXA1 P15	FJ548855	-	V	-	-	-	-	-	-	Q	R	M	D	K	-	-	-	-
	JXA1 P45	FJ548851	-	V	-	-	-	-	-	-	Q	R	M	D	K	-	-	-	-
	JXA1 P70	FJ548852	-	V	-	-	-	-	-	-	Q	R	M	D	K	-	-	-	-
	JXA1 P80	FJ548853	-	V	-	-	-	-	-	-	Q	R	M	D	K	-	-	-	-
	JXA1 P100	KC422725	-	V	-	-	-	-	-	-	Q	R	M	D	K	-	-	-	-
	JXA1 P110	KC422726	-	V	-	-	-	-	-	-	Q	R	M	D	K	-	-	-	-
	JXA1 P120	KC422727	-	V	-	-	-	-	-	-	Q	R	M	D	K	-	-	-	-
	JXA1-P130	KC422728	-	V	-	-	-	-	-	-	Q	R	M	D	K	-	-	-	-
	JXA1-P140	KC422729	-	V	-	-	-	-	-	-	Q	R	M	D	K	-	-	-	-
	JXA1-P150	KC422730	-	V	-	-	-	-	-	-	Q	R	M	D	K	-	-	-	-
	JXA1-P160	KC422731	-	V	-	-	-	-	-	-	Q	R	M	D	K	-	-	-	-
	JXA1-P170	JQ804986	-	V	-	-	-	-	-	-	Q	R	M	D	K	T	-	-	-
JX143	JX143	EU708726	-	-	-	E	-	-	-	-	Q	R	M	D	K	T	-	-	-
	JXM20	GQ499193	-	-	-	K	-	-	-	-	Q	R	M	D	K	T	-	-	-
	JXM40	GQ499194	-	-	-	K	-	-	-	-	Q	R	M	D	K	T	-	-	-
	JXM60	GQ499195	-	-	-	E	-	-	-	-	Q	R	M	D	K	T	-	-	-
	JXM80	GQ499196	-	-	-	E	-	-	-	-	Q	R	M	D	K	T	-	-	-
	JXM100	GQ475526	-	-	-	K	-	-	-	-	Q	R	M	D	K	A	-	-	-
GD	GD	EU825724	-	-	-	-	-	-	-	-	Q	R	M	D	K	-	-	R	-
	GD-P100	GU143913	-	-	-	-	-	-	-	-	Q	R	M	D	K	-	-	W	-
GDQY1	GDQY1	JN387271	-	-	-	-	-	-	-	-	Q	R	M	D	K	-	-	-	-
	GDQY1VP65	JN387272	-	-	-	-	-	-	-	-	Q	R	M	D	K	-	-	-	-
	GDQY1VP80	JN387273	-	-	-	-	-	-	-	-	Q	R	M	D	K	-	-	-	-
	GDQY1VP100	JN387274	-	-	-	-	-	-	-	-	Q	R	M	D	K	-	-	-	-
BJ	BJ	EU825723	-	-	-	-	-	-	-	-	Q	R	M	D	K	-	-	-	-
	BJ-F20	KP890337	-	-	-	-	-	-	-	-	Q	R	M	D	K	-	-	-	-
	BJ-F40	KP890338	-	-	-	-	-	-	-	-	Q	R	M	D	K	-	-	-	-
	BJ-F80	KP890340	-	-	-	-	-	-	-	-	Q	R	M	D	K	-	-	-	-
	BJ-F100	KP890341	-	-	-	-	-	-	-	-	Q	R	M	D	K	-	-	-	-
	BJ-F150	KP890342	-	-	-	-	-	-	-	-	Q	R	M	D	K	-	-	-	-

TP	TP	EU864233	K	-	-	-	H	-	-	-	Q	R	M	D	K	-	-	-	-
	TP_P60	GU232736	K	-	-	-	R	-	-	-	Q	R	M	D	K	-	-	-	-
	TP_P90	GU232737	I	-	-	-	H	-	-	-	Q	R	M	D	K	-	-	-	-
BB0907	BB0907	HQ315835	-	-	-	-	-	E	I	-	Q	R	M	D	K	-	D	-	-
	BB0907-s34	KM453698	-	-	-	-	-	E	V	-	Q	R	M	D	K	-	V	-	-
	BB0907-F44	KM453699	-	-	-	-	-	K	V	-	Q	R	M	D	K	-	D	-	-
Series	Name	NO.	GP2											E					GP3
			50	118	132	168	177	198	204	221	239	250	251	3	9	40	48	62	13
	VR2332	EF536003	F	I	S	M	S	G	H	V	R	I	F	S	D	T	F	P	C
HuN4	HuN4	EF635006	Y	I	S	M	S	G	H	V	R	T	F	S	D	T	L	P	R
	HuN4-F40	-	Y	I	-	-	S	-	-	-	-	T	-	-	D	-	L	S	-
	HuN4-F60	-	Y	I	-	-	S	-	-	-	-	T	-	-	D	-	L	S	-
	HuN4-F112	-	S	V	-	-	A	-	-	-	-	I	-	-	N	-	F	S	-
NT0801	NT0801	HQ315836	S	I	-	-	-	-	-	-	-	-	-	-	D	-	-	-	-
	NT0801-P10	KJ523894	S	I	-	-	-	-	-	-	-	-	-	-	D	-	-	-	-
	NT0801-P30	KJ523895	S	V	-	-	-	-	-	-	-	-	-	-	D	-	-	-	-
	NT0801-P50	KJ523896	S	V	-	-	-	-	-	-	-	-	-	-	Y	-	-	-	-
	NT0801-P80	KJ523897	S	V	-	-	-	-	-	-	-	-	-	-	Y	-	-	-	-
JXA1	JXA1	EF112445	Y	I	-	M	-	-	H	-	-	-	F	S	D	-	L	-	-
	JXA1 P10	FJ548854	Y	I	-	M	-	-	H	-	-	-	F	S	D	-	L	-	-
	JXA1 P15	FJ548855	Y	I	-	M	-	-	H	-	-	-	S	S	D	-	L	-	-
	JXA1 P45	FJ548851	F	I	-	T	-	-	H	-	-	-	S	S	H	-	F	-	-
	JXA1 P70	FJ548852	F	I	-	T	-	-	H	-	-	-	S	S	H	-	F	-	-
	JXA1 P80	FJ548853	F	V	-	T	-	-	H	-	-	-	S	S	H	-	F	-	-
	JXA1 P100	KC422725	F	V	-	T	-	-	H	-	-	-	S	S	H	-	F	-	-
	JXA1 P110	KC422726	F	V	-	M	-	-	N	-	-	-	S	A	H	-	F	-	-
	JXA1 P120	KC422727	F	V	-	M	-	-	N	-	-	-	S	S	H	-	F	-	-
	JXA1-P130	KC422728	F	V	-	M	-	-	N	-	-	-	S	A	H	-	F	-	-
	JXA1-P140	KC422729	F	V	-	M	-	-	N	-	-	-	S	A	H	-	F	-	-
	JXA1-P150	KC422730	F	V	-	M	-	-	N	-	-	-	S	A	H	-	F	-	-
	JXA1-P160	KC422731	F	V	-	M	-	-	N	-	-	-	S	A	H	-	F	-	-

	JXA1-P170	JQ804986	F	V	-	M	-	-	N	-	-	-	S	A	H	-	F	-	-
JX143	JX143	EU708726	-	-	-	-	-	-	-	-	R	-	-	-	-	-	-	-	-
	JXM20	GQ499193	-	-	-	-	-	-	-	-	S	-	-	-	-	-	-	-	-
	JXM40	GQ499194	-	-	-	-	-	-	-	-	S	-	-	-	-	-	-	-	-
	JXM60	GQ499195	-	-	-	-	-	-	-	-	S	-	-	-	-	-	-	-	-
	JXM80	GQ499196	-	-	-	-	-	-	-	-	R	-	-	-	-	-	-	-	-
	JXM100	GQ475526	-	-	-	-	-	-	-	-	S	-	-	-	-	-	-	-	-
GD	GD	EU825724	-	-	-	-	-	-	-	-	-	-	-	-	D	-	-	-	-
	GD-P100	GU143913	-	-	-	-	-	-	-	-	-	-	-	-	N	-	-	-	-
GDQY1	GDQY1	JN387271	Y	I	S	-	-	-	H	-	-	-	-	-	D	-	L	-	-
	GDQY1VP65	JN387272	Y	I	S	-	-	-	H	-	-	-	-	-	N	-	L	-	-
	GDQY1VP80	JN387273	F	V	N	-	-	-	N	-	-	-	-	-	N	-	F	-	-
	GDQY1VP100	JN387274	F	V	N	-	-	-	N	-	-	-	-	-	N	-	F	-	-
BJ	BJ	EU825723	Y	I	S	-	-	-	-	-	-	-	-	-	D	-	L	-	-
	BJ-F20	KP890337	Y	T	S	-	-	-	-	-	-	-	-	-	D	-	L	-	-
	BJ-F40	KP890338	Y	T	S	-	-	-	-	-	-	-	-	-	D	-	L	-	-
	BJ-F80	KP890340	F	T	N	-	-	-	-	-	-	-	-	-	N	-	F	-	-
	BJ-F100	KP890341	F	T	S	-	-	-	-	-	-	-	-	-	N	-	F	-	-
	BJ-F150	KP890342	F	T	N	-	-	-	-	-	-	-	-	-	N	-	F	-	-
TP	TP	EU864233	Y	I	-	-	-	-	-	V	-	T	-	-	D	T	L	-	R
	TP_P60	GU232736	F	V	-	-	-	-	-	I	-	I	-	-	H	S	F	-	H
	TP_P90	GU232737	F	V	-	-	-	-	-	I	-	I	-	-	H	S	F	-	H
BB0907	BB0907	HQ315835	-	-	-	-	-	G	-	-	-	-	-	-	D	-	-	-	-
	BB0907-s34	KM453698	-	-	-	-	-	V	-	-	-	-	-	-	D	-	-	-	-
	BB0907-F44	KM453699	-	-	-	-	-	G	-	-	-	-	-	-	N	-	-	-	-
Series	Name	NO.	GP3																
			31	48	69	71	73	79	111	134	143	165	206	215	216	217	225	226	228
	VR2332	EF536003	T	T	P	R	L	Y	L	R	L	F	N	I	L	R	A	L	S
HuN4	HuN4	EF635006	T	M	P	K	L	H	L	Q	F	F	N	T	S	K	T	S	S
	HuN4-F40	-	-	V	-	-	F	-	-	-	-	-	S	-	-	-	-	-	-
	HuN4-F60	-	-	V	-	-	F	-	-	-	-	-	S	-	-	-	-	-	-

	HuN4-F112	-	-	V	-	-	F	-	-	-	-	-	S	-	-	-	-	-	-
NT0801	NT0801	HQ315836	-	-	-	-	F	-	-	-	S	-	S	-	-	-	-	-	-
	NT0801-P10	KJ523894	-	-	-	-	F	-	-	-	S	-	S	-	-	-	-	-	-
	NT0801-P30	KJ523895	-	-	-	-	F	-	-	-	S	-	S	-	-	-	-	-	-
	NT0801-P50	KJ523896	-	-	-	-	F	-	-	-	L	-	S	-	-	-	-	-	-
	NT0801-P80	KJ523897	-	-	-	-	F	-	-	-	L	-	S	-	-	-	-	-	-
JXA1	JXA1	EF112445	-	-	-	-	F	H	-	-	F	F	S	-	L	-	T	S	-
	JXA1 P10	FJ548854	-	-	-	-	F	H	-	-	F	F	S	-	L	-	T	S	-
	JXA1 P15	FJ548855	-	-	-	-	F	H	-	-	F	F	S	-	L	-	A	S	-
	JXA1 P45	FJ548851	-	-	-	-	F	H	-	-	F	F	S	-	L	-	A	S	-
	JXA1 P70	FJ548852	-	-	-	-	F	N	-	-	F	F	S	-	L	-	A	L	-
	JXA1 P80	FJ548853	-	-	-	-	F	N	-	-	F	F	S	-	L	-	A	L	-
	JXA1 P100	KC422725	-	-	-	-	F	N	-	-	F	F	S	-	L	-	A	S	-
	JXA1 P110	KC422726	-	-	-	-	F	N	-	-	F	F	S	-	L	-	A	S	-
	JXA1 P120	KC422727	-	-	-	-	F	H	-	-	Y	F	S	-	L	-	A	S	-
	JXA1-P130	KC422728	-	-	-	-	F	Y	-	-	F	F	S	-	L	-	A	L	-
	JXA1-P140	KC422729	-	-	-	-	F	Y	-	-	F	F	S	-	L	-	A	S	-
	JXA1-P150	KC422730	-	-	-	-	F	N	-	-	F	F	S	-	L	-	A	L	-
	JXA1-P160	KC422731	-	-	-	-	F	Y	-	-	F	Y	S	-	L	-	A	S	-
	JXA1-P170	JQ804986	-	-	-	-	F	Y	-	-	F	Y	S	-	S	-	A	L	-
JX143	JX143	EU708726	T	-	-	-	F	H	L	-	F	-	S	-	-	-	T	-	-
	JXM20	GQ499193	K	-	-	-	F	H	L	-	F	-	S	-	-	-	A	-	-
	JXM40	GQ499194	K	-	-	-	F	H	L	-	F	-	S	-	-	-	A	-	-
	JXM60	GQ499195	K	-	-	-	F	Y	L	-	L	-	S	-	-	-	A	-	-
	JXM80	GQ499196	T	-	-	-	F	Y	L	-	F	-	S	-	-	-	A	-	-
	JXM100	GQ475526	K	-	-	-	F	Y	S	-	L	-	S	-	-	-	A	-	-
GD	GD	EU825724	-	-	-	-	F	-	-	-	-	-	S	-	-	-	-	S	-
	GD-P100	GU143913	-	-	-	-	F	-	-	-	-	-	S	-	-	-	-	L	-
GDQY1	GDQY1	JN387271	-	-	-	-	F	H	-	-	F	-	S	T	-	-	-	-	-
	GDQY1VP65	JN387272	-	-	-	-	F	Y	-	-	L	-	S	I	-	-	-	-	-
	GDQY1VP80	JN387273	-	-	-	-	F	Y	-	-	L	-	S	T	-	-	-	-	-

	GDQY1VP100	JN387274	-	-	-	-	F	Y	-	-	L	-	S	T	-	-	-	-	-
BJ	BJ	EU825723	-	-	-	-	F	-	-	Q	-	-	S	-	-	-	T	-	-
	BJ-F20	KP890337	-	-	-	-	F	-	-	Q	-	-	S	-	-	-	A	-	-
	BJ-F40	KP890338	-	-	-	-	F	-	-	Q	-	-	S	-	-	-	A	-	-
	BJ-F80	KP890340	-	-	-	-	F	-	-	R	-	-	S	-	-	-	A	-	-
	BJ-F100	KP890341	-	-	-	-	F	-	-	R	-	-	S	-	-	-	A	-	-
	BJ-F150	KP890342	-	-	-	-	F	-	-	R	-	-	S	-	-	-	A	-	-
TP	TP	EU864233	-	-	-	-	F	-	-	-	F	-	S	-	-	K	T	-	F
	TP_P60	GU232736	-	-	-	-	F	-	-	-	S	-	S	-	-	K	A	-	F
	TP_P90	GU232737	-	-	-	-	F	-	-	-	S	-	S	-	-	R	A	-	S
BB0907	BB0907	HQ315835	-	-	P	K	F	H	-	-	-	-	S	-	L	-	-	S	-
	BB0907-s34	KM453698	-	-	S	R	F	H	-	-	-	-	S	-	L	-	-	P	-
	BB0907-F44	KM453699	-	-	P	K	F	Y	-	-	-	-	S	-	L	-	-	S	-
Series	Name	NO.	GP3			GP4													
			238	244	248	14	42	43	44	51	56	66	67	124	129	132	141	160	172
	VR2332	EF536003	A	R	S	C	A	S	F	S	R	I	P	I	V	T	F	E	F
HuN4	HuN4	EF635006	A	R	V	C	S	N	F	S	G	S	S	I	V	T	F	E	F
	HuN4-F40	-	-	-	F	-	-	D	-	-	-	I	P	V	I	-	-	E	-
	HuN4-F60	-	-	-	F	-	-	D	-	-	-	I	P	V	I	-	-	E	-
	HuN4-F112	-	-	-	F	-	-	N	-	-	-	I	P	V	I	-	-	G	-
NT0801	NT0801	HQ315836	-	-	-	-	-	G	-	-	-	-	-	-	-	-	-	-	-
	NT0801-P10	KJ523894	-	-	-	-	-	G	-	-	-	-	-	-	-	-	-	-	-
	NT0801-P30	KJ523895	-	-	-	-	-	G	-	-	-	-	-	-	-	-	-	-	-
	NT0801-P50	KJ523896	-	-	-	-	-	G	-	-	-	-	-	-	-	-	-	-	-
	NT0801-P80	KJ523897	-	-	-	-	-	G	-	-	-	-	-	-	-	-	-	-	-
JXA1	JXA1	EF112445	-	R	F	C	-	D	-	-	-	I	S	I	-	S	-	-	L
	JXA1 P10	FJ548854	-	R	F	C	-	D	-	-	-	I	S	I	-	S	-	-	F
	JXA1 P15	FJ548855	-	R	V	C	-	G	-	-	-	S	S	I	-	T	-	-	F
	JXA1 P45	FJ548851	-	R	V	C	-	G	-	-	-	S	S	I	-	T	-	-	V
	JXA1 P70	FJ548852	-	R	V	C	-	G	-	-	-	S	P	I	-	T	-	-	V
	JXA1 P80	FJ548853	-	R	V	C	-	G	-	-	-	S	S	I	-	T	-	-	V

	JXA1 P100	KC422725	-	R	V	C	-	G	-	-	-	S	S	V	-	T	-	-	V
	JXA1 P110	KC422726	-	R	V	C	-	G	-	-	-	S	S	V	-	T	-	-	F
	JXA1 P120	KC422727	-	Q	V	C	-	G	-	-	-	S	S	V	-	T	-	-	F
	JXA1-P130	KC422728	-	R	V	C	-	G	-	-	-	S	S	V	-	T	-	-	F
	JXA1-P140	KC422729	-	Q	V	C	-	G	-	-	-	S	S	V	-	T	-	-	F
	JXA1-P150	KC422730	-	R	V	C	-	G	-	-	-	S	S	V	-	T	-	-	F
	JXA1-P160	KC422731	-	R	V	Y	-	G	-	-	-	S	S	V	-	T	-	-	F
	JXA1-P170	JQ804986	-	R	V	Y	-	G	-	-	-	S	S	V	-	T	-	-	F
JX143	JX143	EU708726	-	-	-	-	-	D	-	-	-	-	-	-	-	-	F	-	-
	JXM20	GQ499193	-	-	-	-	-	G	-	-	-	-	-	-	-	-	F	-	-
	JXM40	GQ499194	-	-	-	-	-	G	-	-	-	-	-	-	-	-	L	-	-
	JXM60	GQ499195	-	-	-	-	-	G	-	-	-	-	-	-	-	-	F	-	-
	JXM80	GQ499196	-	-	-	-	-	G	-	-	-	-	-	-	-	-	F	-	-
	JXM100	GQ475526	-	-	-	-	-	G	-	-	-	-	-	-	-	-	F	-	-
GD	GD	EU825724	-	-	F	-	-	D	-	-	-	I	-	I	-	-	-	-	-
	GD-P100	GU143913	-	-	F	-	-	N	-	-	-	I	-	T	-	-	-	-	-
GDQY1	GDQY1	JN387271	-	-	F	-	S	D	-	-	-	I	S	-	-	-	-	-	-
	GDQY1VP65	JN387272	-	-	F	-	S	D	-	-	-	I	P	-	-	-	-	-	-
	GDQY1VP80	JN387273	-	-	F	-	S	N	-	-	-	I	S	-	-	-	-	-	-
	GDQY1VP100	JN387274	-	-	F	-	P	N	-	-	-	I	S	-	-	-	-	-	-
BJ	BJ	EU825723	-	-	-	-	-	D	-	G	-	-	-	I	-	-	-	-	-
	BJ-F20	KP890337	-	-	-	-	-	G	-	G	-	-	-	I	-	-	-	-	-
	BJ-F40	KP890338	-	-	-	-	-	G	-	G	-	-	-	V	-	-	-	-	-
	BJ-F80	KP890340	-	-	-	-	-	G	-	S	-	-	-	V	-	-	-	-	-
	BJ-F100	KP890341	-	-	-	-	-	G	-	S	-	-	-	V	-	-	-	-	-
	BJ-F150	KP890342	-	-	-	-	-	G	-	S	-	-	-	V	-	-	-	-	-
TP	TP	EU864233	A	-	F	-	-	D	-	-	G	I	S	-	-	-	-	E	-
	TP_P60	GU232736	S	-	F	-	-	G	-	-	V	I	S	-	-	-	-	G	-
	TP_P90	GU232737	A	-	F	-	-	G	-	-	G	I	P	-	-	-	-	G	-
BB0907	BB0907	HQ315835	-	-	-	-	-	D	F	-	-	-	-	-	-	-	-	-	-
	BB0907-s34	KM453698	-	-	-	-	-	N	S	-	-	-	-	-	-	-	-	-	-

	BB0907-F44	KM453699	-	-	-	-	-	N	F	-	-	-	-	-	-	-	-	-	-

Series	Name	NO.	GP4		GP5a		GP5												
			173	174	2-5	4	11	15	33	34	35	40	52	55	56	59	80	89	102
	VR2332	EF536003	A	I	-	Y	C	L	N	D	S	Q	G	W	L	K	G	D	V
HuN4	HuN4	EF635006	A	I	-	Y	C	L	-	N	N	Q	G	W	L	K	G	D	Y
	HuN4-F40	-	-	-	-	-	-	-	-	N	-	-	-	-	-	-	-	-	-
	HuN4-F60	-	-	-	-	-	-	-	-	N	-	-	-	-	-	-	-	-	-
	HuN4-F112	-	-	-	-	-	-	-	-	D	-	-	-	-	-	-	-	-	-
NT0801	NT0801	HQ315836	-	-	-	-	-	P	-	-	-	R	-	-	-	-	-	-	-
	NT0801-P10	KJ523894	-	-	-	-	-	P	-	-	-	R	-	-	-	-	-	-	-
	NT0801-P30	KJ523895	-	-	-	-	-	P	-	-	-	Q	-	-	-	-	-	-	-
	NT0801-P50	KJ523896	-	-	-	-	-	P	-	-	-	Q	-	-	-	-	-	-	-
	NT0801-P80	KJ523897	-	-	-	-	-	L	-	-	-	Q	-	-	-	-	-	-	-
JXA1	JXA1	EF112445	P	S	-	-	-	-	N	-	-	-	-	-	-	K	-	D	-
	JXA1 P10	FJ548854	A	I	-	-	-	-	N	-	-	-	-	-	-	K	-	D	-
	JXA1 P15	FJ548855	A	I	-	-	-	-	N	-	-	-	-	-	-	K	-	D	-
	JXA1 P45	FJ548851	A	I	-	-	-	-	N	-	-	-	-	-	-	K	-	D	-
	JXA1 P70	FJ548852	A	I	INS	-	-	-	N	-	-	-	-	-	-	N	-	D	-
	JXA1 P80	FJ548853	A	I	INS	-	-	-	N	-	-	-	-	-	-	N	-	G	-
	JXA1 P100	KC422725	A	I	-	-	-	-	N	-	-	-	-	-	-	N	-	D	-
	JXA1 P110	KC422726	A	I	-	-	-	-	D	-	-	-	-	-	-	N	-	D	-
	JXA1 P120	KC422727	A	I	-	-	-	-	D	-	-	-	-	-	-	N	-	D	-
	JXA1-P130	KC422728	A	I	-	-	-	-	D	-	-	-	-	-	-	N	-	D	-
	JXA1-P140	KC422729	A	I	-	-	-	-	D	-	-	-	-	-	-	N	-	D	-
	JXA1-P150	KC422730	A	I	-	-	-	-	D	-	-	-	-	-	-	N	-	D	-
	JXA1-P160	KC422731	A	I	-	-	-	-	D	-	-	-	-	-	-	N	-	D	-
	JXA1-P170	JQ804986	A	I	-	-	-	-	D	-	-	-	-	-	-	N	-	D	-
JX143	JX143	EU708726	-	-	-	-	-	-	-	-	-	-	-	-	-	-	-	-	-
	JXM20	GQ499193	-	-	-	-	-	-	-	-	-	-	-	-	-	-	-	-	-
	JXM40	GQ499194	-	-	-	-	-	-	-	-	-	-	-	-	-	-	-	-	-
	JXM60	GQ499195	-	-	-	-	-	-	-	-	-	-	-	-	-	-	-	-	-

	JXM80	GQ499196	-	-	-	-	-	-	-	-	-	-	-	-	-	-	-	-	-
	JXM100	GQ475526	-	-	-	-	-	-	-	-	-	-	-	-	-	-	-	-	-
GD	GD	EU825724	-	-	-	-	-	-	-	-	-	-	-	-	-	-	-	-	-
	GD-P100	GU143913	-	-	-	-	-	-	-	-	-	-	-	-	-	-	-	-	-
GDQY1	GDQY1	JN387271	-	-	-	Y	-	-	-	-	-	-	G	W	L	-	G	-	-
	GDQY1VP65	JN387272	-	-	-	N	-	-	-	-	-	-	G	W	L	-	V	-	-
	GDQY1VP80	JN387273	-	-	-	Y	-	-	-	-	-	-	G	F	L	-	G	-	-
	GDQY1VP100	JN387274	-	-	-	Y	-	-	-	-	-	-	V	W	P	-	V	-	-
BJ	BJ	EU825723	-	-	-	-	Y	-	-	-	-	-	-	-	-	-	-	-	-
	BJ-F20	KP890337	-	-	-	-	Y	-	-	-	-	-	-	-	-	-	-	-	-
	BJ-F40	KP890338	-	-	-	-	C	-	-	-	-	-	-	-	-	-	-	-	-
	BJ-F80	KP890340	-	-	-	-	C	-	-	-	-	-	-	-	-	-	-	-	-
	BJ-F100	KP890341	-	-	-	-	C	-	-	-	-	-	-	-	-	-	-	-	-
	BJ-F150	KP890342	-	-	-	-	C	-	-	-	-	-	-	-	-	-	-	-	-
TP	TP	EU864233	-	-	-	-	-	-	-	-	-	-	-	-	-	-	-	-	-
	TP_P60	GU232736	-	-	-	-	-	-	-	-	-	-	-	-	-	-	-	-	-
	TP_P90	GU232737	-	-	-	-	-	-	-	-	-	-	-	-	-	-	-	-	-
BB0907	BB0907	HQ315835	-	-	-	-	-	-	-	-	N	-	-	-	-	-	-	-	Y
	BB0907-s34	KM453698	-	-	-	-	-	-	-	-	N	-	-	-	-	-	-	-	C
	BB0907-F44	KM453699	-	-	-	-	-	-	-	-	D	-	-	-	-	-	-	-	Y
Series	Name	NO.	GP5							M							N	3UTR	
			104	134	159	164	170	173	196	63	93	107	128	129	146	164	11	3	36
	VR2332	EF536003	G	W	V	R	E	L	Q	A	K	K	N	D	T	Q	R	C	T
HuN4	HuN4	EF635006	G	W	V	G	K	L	Q	V	K	R	N	D	T	Q	R	T	T
	HuN4-F40	-	-	-	-	-	E	-	R	V	-	K	-	-	-	-	K	C	-
	HuN4-F60	-	-	-	-	-	E	-	R	A	-	K	-	-	-	-	K	C	-
	HuN4-F112	-	-	-	-	-	E	-	R	A	-	K	-	-	-	-	K	C	-
NT0801	NT0801	HQ315836	-	-	-	-	G	-	-	-	-	K	-	-	-	-	K	C	-
	NT0801-P10	KJ523894	-	-	-	-	G	-	-	-	-	K	-	-	-	-	K	C	-
	NT0801-P30	KJ523895	-	-	-	-	G	-	-	-	-	K	-	-	-	-	K	C	-
	NT0801-P50	KJ523896	-	-	-	-	R	-	-	-	-	K	-	-	-	-	K	C	-

	NT0801-P80	KJ523897	-	-	-	-	R	-	-	-	-	K	-	-	-	-	K	C	-
JXA1	JXA1	EF112445	-	-	-	G	-	-	L	-	-	K	-	-	T	-	K	C	-
	JXA1 P10	FJ548854	-	-	-	G	-	-	Q	-	-	K	-	-	T	-	K	C	-
	JXA1 P15	FJ548855	-	-	-	G	-	-	Q	-	-	K	-	-	T	-	K	C	-
	JXA1 P45	FJ548851	-	-	-	R	-	-	L	-	-	K	-	-	T	-	K	C	-
	JXA1 P70	FJ548852	-	-	-	R	-	-	L	-	-	K	-	-	T	-	K	C	-
	JXA1 P80	FJ548853	-	-	-	R	-	-	L	-	-	K	-	-	A	-	K	C	-
	JXA1 P100	KC422725	-	-	-	R	-	-	L	-	-	K	-	-	T	-	K	C	-
	JXA1 P110	KC422726	-	-	-	R	-	-	L	-	-	K	-	-	T	-	K	C	-
	JXA1 P120	KC422727	-	-	-	R	-	-	L	-	-	K	-	-	T	-	K	C	-
	JXA1-P130	KC422728	-	-	-	R	-	-	L	-	-	K	-	-	T	-	K	C	-
	JXA1-P140	KC422729	-	-	-	R	-	-	L	-	-	K	-	-	T	-	K	C	-
	JXA1-P150	KC422730	-	-	-	R	-	-	L	-	-	K	-	-	T	-	N	C	-
	JXA1-P160	KC422731	-	-	-	R	-	-	L	-	-	K	-	-	T	-	N	C	-
	JXA1-P170	JQ804986	-	-	-	R	-	-	L	-	-	K	-	-	T	-	N	C	-
JX143	JX143	EU708726	-	-	-	-	-	-	-	-	-	K	-	-	-	Q	K	C	-
	JXM20	GQ499193	-	-	-	-	-	-	-	-	-	K	-	-	-	Q	K	C	-
	JXM40	GQ499194	-	-	-	-	-	-	-	-	-	K	-	-	-	Q	K	C	-
	JXM60	GQ499195	-	-	-	-	-	-	-	-	-	K	-	-	-	R	K	C	-
	JXM80	GQ499196	-	-	-	-	-	-	-	-	-	K	-	-	-	R	K	C	-
	JXM100	GQ475526	-	-	-	-	-	-	-	-	-	K	-	-	-	R	K	C	-
GD	GD	EU825724	-	-	-	-	-	-	Q	-	K	K	-	-	-	-	K	C	-
	GD-P100	GU143913	-	-	-	-	-	-	R	-	R	K	-	-	-	-	K	C	-
GDQY1	GDQY1	JN387271	-	-	-	-	-	-	Q	-	-	K	-	-	-	-	K	C	-
	GDQY1VP65	JN387272	-	-	-	-	-	-	R	-	-	K	-	-	-	-	K	C	-
	GDQY1VP80	JN387273	-	-	-	-	-	-	R	-	-	K	-	-	-	-	K	C	-
	GDQY1VP100	JN387274	-	-	-	-	-	-	R	-	-	K	-	-	-	-	K	C	-
BJ	BJ	EU825723	-	-	V	-	-	-	Q	-	-	K	N	D	-	-	K	C	-
	BJ-F20	KP890337	-	-	V	-	-	-	R	-	-	K	N	D	-	-	K	C	-
	BJ-F40	KP890338	-	-	V	-	-	-	R	-	-	K	N	D	-	-	K	C	-
	BJ-F80	KP890340	-	-	I	-	-	-	R	-	-	K	N	D	-	-	K	C	-

	BJ-F100	KP890341	-	-	V	-	-	-	R	-	-	K	T	H	-	-	K	C	-
	BJ-F150	KP890342	-	-	V	-	-	-	R	-	-	K	T	H	-	-	K	C	-
TP	TP	EU864233	-	W	-	-	-	L	-	V	-	K	-	-	-	-	K	C	C
	TP_P60	GU232736	-	W	-	-	-	R	-	V	-	K	-	-	-	-	K	C	T
	TP_P90	GU232737	-	R	-	-	-	R	-	A	-	K	-	-	-	-	K	C	T
BB0907	BB0907	HQ315835	G	-	-	-	-	-	-	-	-	K	-	-	-	-	K	T	-
	BB0907-s34	KM453698	R	-	-	-	-	-	-	-	-	K	-	-	-	-	K	T	-
	BB0907-F44	KM453699	G	-	-	-	-	-	-	-	-	K	-	-	-	-	K	T	-
Series	Name	NO.	3UTR																
			49	69	74	83	102	109	118	146	149	170							
	VR2332	EF536003	G	G	A	G	T	C	C	T	G	G							
HuN4	HuN4	EF635006	A	A	A	G	T	C	C	T	A	G							
	HuN4-F40	-	G	G	-	-	-	-	-	-	-	-							
	HuN4-F60	-	G	G	-	-	-	-	-	-	-	-							
	HuN4-F112	-	G	G	-	-	-	-	-	-	-	-							
NT0801	NT0801	HQ315836	-	G	-	-	-	-	-	-	-	G							
	NT0801-P10	KJ523894	-	G	-	-	-	-	-	-	-	G							
	NT0801-P30	KJ523895	-	G	-	-	-	-	-	-	-	G							
	NT0801-P50	KJ523896	-	G	-	-	-	-	-	-	-	G							
	NT0801-P80	KJ523897	-	G	-	-	-	-	-	-	-	A							
JXA1	JXA1	EF112445	G	G	A	-	-	C	-	T	-	-							
	JXA1 P10	FJ548854	G	G	A	-	-	C	-	T	-	-							
	JXA1 P15	FJ548855	G	G	A	-	-	C	-	T	-	-							
	JXA1 P45	FJ548851	G	G	G	-	-	C	-	C	-	-							
	JXA1 P70	FJ548852	G	G	A	-	-	C	-	C	-	-							
	JXA1 P80	FJ548853	G	G	A	-	-	C	-	C	-	-							
	JXA1 P100	KC422725	G	G	A	-	-	C	-	C	-	-							
	JXA1 P110	KC422726	G	G	A	-	-	C	-	C	-	-							
	JXA1 P120	KC422727	G	G	A	-	-	T	-	C	-	-							
	JXA1-P130	KC422728	G	G	A	-	-	C	-	C	-	-							
	JXA1-P140	KC422729	G	G	A	-	-	C	-	C	-	-							

	JXA1-P150	KC422730	G	G	A	-	-	C	-	C	-	-	
	JXA1-P160	KC422731	G	G	A	-	-	C	-	C	-	-	
	JXA1-P170	JQ804986	G	G	A	-	-	C	-	C	-	-	
JX143	JX143	EU708726	G	G	-	-	-	-	-	-	-	-	
	JXM20	GQ499193	G	G	-	-	-	-	-	-	-	-	
	JXM40	GQ499194	G	G	-	-	-	-	-	-	-	-	
	JXM60	GQ499195	G	G	-	-	-	-	-	-	-	-	
	JXM80	GQ499196	G	G	-	-	-	-	-	-	-	-	
	JXM100	GQ475526	G	G	-	-	-	-	-	-	-	-	
GD	GD	EU825724	G	G	-	-	-	-	-	-	-	-	
	GD-P100	GU143913	G	G	-	-	-	-	-	-	-	-	
GDQY1	GDQY1	JN387271	G	G	-	-	T	-	-	-	-	-	
	GDQY1VP65	JN387272	G	G	-	-	T	-	-	-	-	-	
	GDQY1VP80	JN387273	G	G	-	-	T	-	-	-	-	-	
	GDQY1VP100	JN387274	G	G	-	-	A	-	-	-	-	-	
BJ	BJ	EU825723	G	G	-	G	-	-	-	-	-	-	
	BJ-F20	KP890337	G	G	-	G	-	-	-	-	-	-	
	BJ-F40	KP890338	G	G	-	G	-	-	-	-	-	-	
	BJ-F80	KP890340	G	G	-	C	-	-	-	-	-	-	
	BJ-F100	KP890341	G	G	-	G	-	-	-	-	-	-	
	BJ-F150	KP890342	G	G	-	G	-	-	-	-	-	-	
TP	TP	EU864233	G	G	-	-	-	-	-	-	A	-	
	TP_P60	GU232736	G	G	-	-	-	-	-	-	G	-	
	TP_P90	GU232737	G	G	-	-	-	-	-	-	G	-	
BB0907	BB0907	HQ315835	G	G	-	-	-	-	C	-	-	-	
	BB0907-s34	KM453698	G	G	-	-	-	-	T	-	-	-	
	BB0907-F44	KM453699	G	G	-	-	-	-	C	-	-	-	

PRRSV VR2332 was used as a reference for the position of amino acid or nucleotide sites.
